# A high-quality *Bougainvillea* genome provides new insights into evolutionary history and pigment biosynthetic pathways in the Caryophyllales

**DOI:** 10.1093/hr/uhad124

**Published:** 2023-06-13

**Authors:** Lan Lan, Huiqi Zhao, Suxia Xu, Shenglong Kan, Xiaoni Zhang, Weichao Liu, Xuezhu Liao, Luke R Tembrock, Yonglin Ren, Wayne Reeve, Jun Yang, Zhiqiang Wu

**Affiliations:** Shenzhen Branch, Guangdong Laboratory of Lingnan Modern Agriculture, Genome Analysis Laboratory of the Ministry of Agriculture and Rural Affairs, Agricultural Genomics Institute at Shenzhen, Chinese Academy of Agricultural Sciences, Shenzhen, 518120, China; School of Medical, Molecularand Forensic Sciences, Murdoch University, 6150, Western Australia, 90 South Street, Murdoch, Australia; Kunpeng Institute of Modern Agriculture at Foshan, Shenzhen Branch, Guangdong Laboratory of Lingnan Modern Agriculture, Agricultural Genomics Institute at Shenzhen, Chinese Academy of Agricultural Sciences, Shenzhen 518124, China; Sanya Institute, Hainan Academy of Agricultural Sciences, Sanya, 572025, China; Institute of Tropical Horticulture Research, Hainan Academy of Agricultural Sciences, Haikou, 571100, China; Fujian Key Laboratory of Subtropical Plant Physiology & Biochemistry, Fujian Institute of Subtropical Botany, Xiamen, 361006, China; Kunpeng Institute of Modern Agriculture at Foshan, Shenzhen Branch, Guangdong Laboratory of Lingnan Modern Agriculture, Agricultural Genomics Institute at Shenzhen, Chinese Academy of Agricultural Sciences, Shenzhen 518124, China; Shenzhen Branch, Guangdong Laboratory of Lingnan Modern Agriculture, Genome Analysis Laboratory of the Ministry of Agriculture and Rural Affairs, Agricultural Genomics Institute at Shenzhen, Chinese Academy of Agricultural Sciences, Shenzhen, 518120, China; Kunpeng Institute of Modern Agriculture at Foshan, Shenzhen Branch, Guangdong Laboratory of Lingnan Modern Agriculture, Agricultural Genomics Institute at Shenzhen, Chinese Academy of Agricultural Sciences, Shenzhen 518124, China; Kunpeng Institute of Modern Agriculture at Foshan, Shenzhen Branch, Guangdong Laboratory of Lingnan Modern Agriculture, Agricultural Genomics Institute at Shenzhen, Chinese Academy of Agricultural Sciences, Shenzhen 518124, China; Key Laboratory of Horticultural Plant Biology, College of Horticulture and Forestry Sciences, Huazhong Agricultural University, Wuhan, 430070, China; Shenzhen Branch, Guangdong Laboratory of Lingnan Modern Agriculture, Genome Analysis Laboratory of the Ministry of Agriculture and Rural Affairs, Agricultural Genomics Institute at Shenzhen, Chinese Academy of Agricultural Sciences, Shenzhen, 518120, China; Kunpeng Institute of Modern Agriculture at Foshan, Shenzhen Branch, Guangdong Laboratory of Lingnan Modern Agriculture, Agricultural Genomics Institute at Shenzhen, Chinese Academy of Agricultural Sciences, Shenzhen 518124, China; Department of Agricultural Biology, Colorado State University, Fort Collins, CO, 80523, USA; School of Medical, Molecularand Forensic Sciences, Murdoch University, 6150, Western Australia, 90 South Street, Murdoch, Australia; School of Medical, Molecularand Forensic Sciences, Murdoch University, 6150, Western Australia, 90 South Street, Murdoch, Australia; Sanya Institute, Hainan Academy of Agricultural Sciences, Sanya, 572025, China; Institute of Tropical Horticulture Research, Hainan Academy of Agricultural Sciences, Haikou, 571100, China; Shenzhen Branch, Guangdong Laboratory of Lingnan Modern Agriculture, Genome Analysis Laboratory of the Ministry of Agriculture and Rural Affairs, Agricultural Genomics Institute at Shenzhen, Chinese Academy of Agricultural Sciences, Shenzhen, 518120, China; Kunpeng Institute of Modern Agriculture at Foshan, Shenzhen Branch, Guangdong Laboratory of Lingnan Modern Agriculture, Agricultural Genomics Institute at Shenzhen, Chinese Academy of Agricultural Sciences, Shenzhen 518124, China

## Abstract

*Bougainvillea* is a perennial ornamental shrub that is highly regarded in ornamental horticulture around the world. However, the absence of genome data limits our understanding of the pathways involved in bract coloration and breeding. Here, we report a chromosome-level assembly of the giga-genome of *Bougainvillea × buttiana* ‘Mrs Butt’, a cultivar thought to be the origin of many other *Bougainvillea* cultivars. The assembled genome is ~5 Gb with a scaffold N50 of 151 756 278 bp and contains 86 572 genes which have undergone recent whole-genome duplication. We confirmed that multiple rounds of whole-genome multiplication have occurred in the evolutionary history of the Caryophyllales, reconstructed the relationship in the Caryophyllales at whole genome level, and found discordance between species and gene trees as the result of complex introgression events. We investigated betalain and anthocyanin biosynthetic pathways and found instances of independent evolutionary innovations in the nine different Caryophyllales species. To explore the potential formation mechanism of diverse bract colors in *Bougainvillea*, we analyzed the genes involved in betalain and anthocyanin biosynthesis and found extremely low expression of *ANS* and *DFR* genes in all cultivars, which may limit anthocyanin biosynthesis. Our findings indicate that the expression pattern of the betalain biosynthetic pathway did not directly correlate with bract color, and a higher expression level in the betalain biosynthetic pathway is required for colored bracts. This improved understanding of the correlation between gene expression and bract color allows plant breeding outcomes to be predicted with greater certainty.

## Introduction

Caryophyllales is a large and diverse clade of angiosperms containing nearly 40 families, 749 genera, and 12 500 species [32]. The early branching history of Caryophyllales is characterized by multiple nested, rapid radiations that have been difficult to reconstruct using standard phylogenetic loci [47, 73, 86]. Species of Caryophyllales are found on every continent, growing in all terrestrial and many aquatic habitats [39]. The Caryophyllales order includes many economically important species, such as beet (*Beta vulgaris*), carnation (*Dianthus caryophyllus*), dragonfruit (*Selenicereus* sp.), spinach (*Spinacia oleracea*), and *Bougainvillea*.

**Figure 1:**
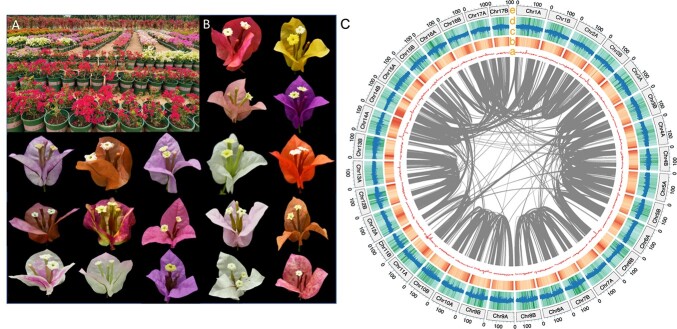
(A) Different *Bougainvillea* cultivars. (B) Blooming stage of different *Bougainvillea* cultivars. (C) Circos plot of BTFR genome. (a) Distribution of GC content; (b) gene density heat map among different chromosomes; (c) Copia transposed element density bar plot; (d) Gypsy transposed element density bar plot; (e) chromosome length. The heat maps in (c) and (d) illustrate the total repeat content density, and the inner lines represent the syntenic links. All the densities were calculated in windows of 2 Mb.

*Bougainvillea* is native to South America and is a highly regarded ornamental plant species throughout the world. Some species produce phytochemicals with antimicrobial activity and thus have both ornamental and prophylactic value [1, 26]. Furthermore, *Bougainvillea* species are drought-tolerant, with a range of ecotypes including spiny trees, shrubs, and vines that enable plants to adapt to different geographical areas [17, 71]. One of the most important ornamental traits of *Bougainvillea* is the brilliant bract color, which is thought to occur as the result of betalain accumulation (Fig. 1A and B). Numerous studies have shown that betalain and anthocyanin pigments cannot coexist within the same plant, suggesting an antagonistic relationship [84, 85]. Betalains comprise two classes of compounds: betaxanthins and betacyanins, which give rise to yellow and violet bracts, respectively [29, 30]. The betalains could be beneficial for photoprotection and attraction of animal pollinators and dispersers, and confer tolerance to drought and salinity [43]. Furthermore, betalains are used as commercial food colorants and additives [29]. Analysis of transcriptome data suggests that loss of genes caused the anthocyanin-producing species to lose the ability to produce betalains [6]. However, the relationship between betalain accumulation and *Bougainvillea* phenotype is not fully understood. Comparative genomic studies are needed to better understand the evolution of genetic architecture in pigment biosynthesis in this genus. The diverse coloration found in different *Bougainvillea* cultivars provides an excellent opportunity to study the evolution of the betalain biosynthetic pathway across the Caryophyllales, applying comparative genomics.

*Bougainvillea × buttiana* ‘Mrs Butt’ was first discovered in 1910 by Mrs Clara Butt, who was surprised by the deep red color of the bracts [90]. For a long time, *B. × buttiana* ‘Mrs Butt’ was regarded as an independent species; however, subsequent research demonstrated that it may be the result of crossbreeding of a *Bougainvillea* from southern Brazil with a *Bougainvillea* from the Northern Andes, Peru. Nowadays, there are almost 500 cultivars distributed throughout the world [53]. Most of these originated from *B. × buttiana* ‘Mrs Butt’ through bud sports, mutation, and inter- and intra-species crossbreeding [17], its red color providing numerous possibilities to enrich bract color.

Given the importance of the *Bougainvillea* species, analysis of this cultivar’s genomic information to understand its evolutionary history and genetic improvement is urgently needed. Phylogenomic studies have focused on understanding the evolutionary basis of a wide range of groups and species, including *Chloranthus sessilifolius* [34], the asterids [99], butterflies [23], the Persian walnut [97], *Drosophila* species [83], and ruminants [13]. Despite the use of whole-genome sequencing data in the above studies, discordance between gene trees was found due to biological factors such as incomplete lineage sorting (ILS) and introgression [20]. Phylogenomic studies using transcriptomic data revealed that several deep nodes were poorly supported [50] and that discordance between gene trees and species trees was widespread in the Caryophyllales [86]. Plastid data have also been used to resolve conflict in the phylogenetic relationships that have emerged from the nuclear data [95]. Despite our improved understanding of the evolutionary relationships within the Caryophyllales, a great deal more remains to be resolved, such as the extent to which ILS and introgression have shaped evolutionary patterns, especially as they pertain to Nyctaginaceae, which has historically been placed in multiple different groupings within the Caryophyllales [5, 16, 95]. The Nyctaginaceae genera (including *Bougainvillea*) present an additional challenge for lineage resolution given the proliferation of the many known cultivars and the difficulty in separating them [77]. The genome data could provide a means to directly distinguish different cultivars. Cultivated *Bougainvillea* were domesticated from the wild ancestor, and there is a need to trace the breeding pathways to guide the development of future cultivars [41]. Thus a high-quality *Bougainvillea* genome sequence is crucial to explain diversity traits due to natural and human selection.

Here, to explore the evolutionary history and bract color formation of *Bougainvillea* species, we assembled a high-quality genome of *B. × buttiana* ‘Mrs Butt’ (hereafter BTFR), which experienced a recent whole-genome duplication (WGD) event with giga-genome size nearing 5 Gb. We used large-scale genomes to explore the phylogeny and evolutionary history of *Bougainvillea* and other species within the Caryophyllales. We demonstrated that introgression is the main source of the discordance between gene trees and species tree. In addition, we analyzed the evolutionary results of the betalain and anthocyanin biosynthetic pathways, and detected different evolutionary patterns of the pathways among nine Caryophyllales genomes. By studying the different cultivars with different bract colors in *Bougainvillea*, we explored the mechanism underlying different colors using expression levels of pigment biosynthetic genes. Overall, our results revealed the complex evolutionary history of the genome and pigment biosynthesis pathways among the nine Caryophyllales genomes and provided greater understanding of the bract color formation mechanism.

## Results

### General features of the BTFR genome

We used 65.37 Gb of HiFi data and 262 Gb of Hi-C data for genome assembly. Using the assembly pipeline described in the Materials and methods section, the final genome size was 5 098 937 527 bp, which is close to the predicted genome size of 5065.6 Mb (Supplementary Data Fig. S1). Through karyotyping (Supplementary Data Fig. S2), we confirmed that the BTFR contains 34 chromosomes in each haplotype (2*n* = 2*x* = 68), and the genome was scaffolded into 34 pseudochromosomes (Fig. 1C, Supplementary Data Fig. S3) with an anchoring ratio of 99.07% and N50 of 151 756 278 bp (Table 1). The BTFR genome had a high complete score of 97.6% from Benchmarking Universal Single-Copy Orthologs (BUSCO) (Supplementary Data Fig. S4), and the *k*-mer spectrum showed that errors were absent in the assembly while the unique content was entirely in the assembly (Supplementary Data Fig. S5). The CC ratio was 138.65. The evaluation results demonstrated a high-quality assembly of the BTFR genome. Furthermore, we detected a high percentage of duplicated gene sets (92.9%) (Supplementary Data Fig. S4), which may support recent WGD events of the BTFR.

**Table 1:** Summary of BTFR genome assembly and annotation.

**Genome feaures**	**Value**
Genome size	5098.9 Mb
N50 length (contigs)	3 076 239 bp
Total contig number	4714
N50 length (scaffold)	151 756 278 bp
Total scaffold numbers	248
Anchor ratio	99.07%
Predicted genes	86 572
Average coding sequence length	924 bp
Average intron length	1412.43 bp
Functionally annotated	65 318 (75.44%)

Through the annotation pipeline, we predicted 86 572 genes with the BUSCO complete score of 95.5%, of which 84.0% were duplicated (Supplementary Data Fig. S6). There were 65 318 predicted genes annotated by different databases (Table 1, Supplementary Data Table S1). Indicates a high quality gene prediction result for BTFR, making it applicable to downstream analyses. Comparing the average gene length, exon length, and intron length, we found that BTFR contained the third longest average genes and the second longest average intron length, but the shortest average exon length when compared with other Caryophyllales genomes (Supplementary Data Table S2). However, the total distribution pattern of gene, exon, and intron length indicated that the available Caryophyllales genomes are similar (Supplementary Data Fig. S7).

We obtained 4 057 392 844 bp of repeat content in BTFR (Supplementary Data Table S3), which account for 79.61% of the total genome length. Compared with other Caryophyllales genomes, we found that BTFR encompassed the largest number of repeats, which could be the main factor contributing to its giga-genome (Supplementary Data Table S4). Long terminal repeats (LTRs) account for 59.7% of the BTFR genome, and the LTR burst occurred near 0.8 Mya (Supplementary Data Fig. S8), which means the genome size expansion may have happened recently.

### Whole-genome multiplication events in Caryophyllales

**Figure 2:**
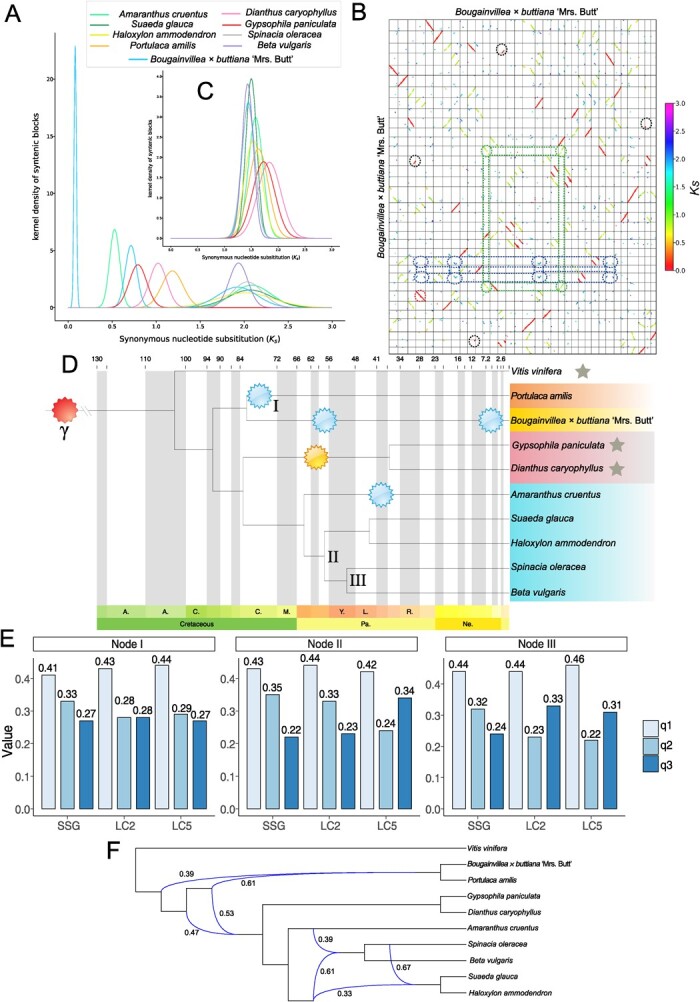
WGD and phylogenomic analysis results. (A) Distribution of *K*_s_, which represents the Gaussian fit of the raw *K*_s_ counts from paralogs. (B) Synteny blocks of the BTFR genome. The axes refer to different chromosomes, the red circle represents the recent WGD event, the green circle represents the WGT event, the blue circle represents the γ event, and the black circle represents the rearrangement events after the recent WGD. (C) Distribution of *K*_s_, which represents the Gaussian fit of the raw *K*_s_ counts from orthologs between *V. vinifera* and the nine Caryophyllales species. (D) Phylogenetic tree generated based on 10 species’ LC2 genes by supermatrix sequences. All bootstrap values were 100, the red symbols in the tree represents the γ event, the yellow symbols in the tree represent the WGT event, the blue symbols in the tree represent the WGD event, the gray stars represent the species producing the anthocyanins, the blue highlight indicates species belonging to the Amaranthaceae, the orange highlight indicates species belonging to *Portulaca*, the pink highlight indicates species belonging to Caryophyllaceae, and the yellow highlight indicates species belong to Nyctaginaceae. (E) Proportions of gene trees with different topologies. Focal internal branches are marked I, II, and III. q1, q2, and q3 indicate the quartet support for the three alternative topologies. (F) Phylogenetic network. Numerical values next to curved branches indicate inheritance probabilities for each hybrid node.

For BTFR, we confirmed that it experienced a recent WGD event and two whole-genome triplication (WGT) events (Fig. 2A and B). The recent WGD event was further supported by the high duplicate score seen in our genome assembly and structure annotation evaluation results, and the collinearity analysis results based on genome data (Supplementary Data Fig. S9). In addition to the γ event, we detected a WGD in *Portulaca amilis* (Fig. 2A, Supplementary Data Fig. S10); a WGT in *D. caryophyllus* and *Gypsophila paniculata* (Supplementary Data Figs S11 and S12), and a WGD in *Amaranthus cruentus* (Supplementary Data Fig. S13). In *Suaeda glauca*, *Haloxylon ammodendron*, *Spinacea oleracea*, and *Beta vulgaris* only the γ duplication event was detectable (Fig. 2A, Supplementary Data Figs S14–S17).

We further confirmed the depth of collinearity regions between *Vitis vinifera* to determine the types of multiplication events. A homologous region showed a 2:1 syntenic relationship between *P. amilis* and *V. vinifera* (Supplementary Data Fig. S18). A 3:1 syntenic relationship was found between *D. caryophyllus* and *G. paniculata*. Comparison with *V. vinifera* (Supplementary Data Figs S19 and S20) indicated that *P. amilis* underwent both a γ event and a WGD event, while *D. caryophyllus* and *G. paniculata* both experienced a γ event and a WGT event. A 3:1 syntenic relationship between a single chromosome of *Bougainvillea* and *V. vinifera* (Supplementary Data Fig. S21) suggest both a WGT and a more recent WGD event, given the existence of duplicate chromosomes as well as the γ event. All other species had a 1:1 syntenic relationship with *V. vinifera* (Supplementary Data Figs S22–S25), suggesting retention of the single γ event only.

The peak synonymous substitutions per synonymous site (*K*_s_) value obtained from the WGDI ‘-pf’ (Supplementary Data Figs S26 and S27) was used to calculate the number of substitutions per synonymous site per year (*μ*) with the formula *μ* = *K*_s_/[2 × (divergence time with *V. vinifera*)] (Fig. 2C) [3]. We found that *B. vulgaris* had the lowest substitution rate (5.97E−09), while *D. caryophyllus* (7.65E−09) had the highest substitution rate (Supplementary Data Table S5), meaning *B. vulgaris* had the slowest rates of molecular evolution on average while *D. caryophyllus* had the fastest rates on average. Also, according to the *μ* = *K*_s_/(2 × divergence time) calculation we were able to estimate the date of different duplication events (Fig. 2D, Supplementary Data Table S5). These results for the inference of WGD support a complex evolutionary history among species of Caryophyllales.

### Contribution of introgression to discordant phylogenetic relationships within the Caryophyllales

From single-copy genes (SCGs), low-copy-2 (LC2) genes, low-copy-5 (LC5) genes and soft-single-copy (SSC) genes, highly supported species trees were obtained through maximum-likelihood (ML) analysis of the supermatrix sequences (Fig. 2D, Supplementary Data Fig. S27A, C, E, and G). Every node indicated that the species relationship was robust, and the topologies were very similar, based on the plastid data [95]. We applied coalescent-based phylogenetic analysis by ASTRAL using the single gene trees from the datasets of SSC, LC2, LC5, and SCGs. Although the phylogenetic topology of concatenated trees was consistent, we found that the internal branches had different support evidence in some nodes (Supplementary Data Fig. S28B, D, F and H). These results concur with previous research findings on the transcriptome phylogeny of other Caryophyllales species [86]. In nodes I, II, and III, the value of q1, q2 and q3 generated by ASTRAL exhibited alternative topologies (Fig. 2E), and different datasets generated a similar pattern, suggesting that the gene trees yielded random topologies.

The results suggest that the ancestor of *Bougainvillea* and *P. amilis* split from the ancestor of Caryophyllales in the Turonian age, nearly 81 Mya (Fig. 2D). Subsequently, *P. amilis* and *Bougainvillea* experienced WGD and WGT events, ~78.2 and 59 Mya, respectively. In addition, *Bougainvillea* experienced a recent individual WGD event. The ancestor of Caryophyllaceae was divided in the Santonian age (nearly 83 mya), and *D. caryophyllus* and *G. paniculata* were divided into two individual species after a WGT event. As for the Amaranthaceae, apart from *A. cruentus*, which experienced a WGD event, none of the species have experienced any further whole-genome multiplication events.

**Figure 3:**
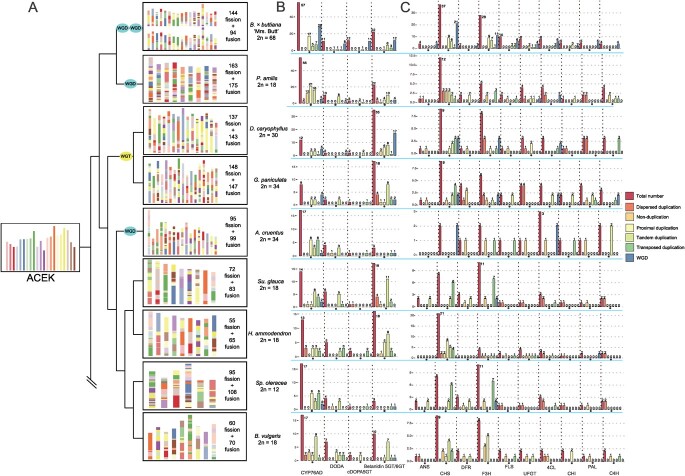
Karyotype analysis and genes in pigment biosynthesis pathways. (A) Different colors represent different ancestor chromosomes from the ACEK, and show that the modern karyotype of different species experienced different evolutionary and recombination events. (B) Genes involved in the betalain biosynthetic pathway and their duplication types. (C) Genes involved in the anthocyanin biosynthetic pathway and their duplication types.

Either ILS or introgression could have contributed to these discordant topologies. The quantifying introgression via branch lengths (QuIBL) program was used to evaluate whether ILS is the prime explanation of the discordance between the species tree and the gene trees across the Caryophyllales. We used 1731 gene trees from LC2 groups and 440 gene trees from SCGs which have been filtered. The Bayesian Information Criterion (BIC) test indicated that the phylogenetic discordances were mostly caused by introgression + ILS (Supplementary Data Tables S6 and S7). For example, in LC2 several discordances were caused by ILS, and only 14 triplets were affected by ILS, accounting for 28% of all the triplets.

A variant of phylogenetic network analysis was further applied according to the 1975 LC2 groups which were filtered by TreeShrink. The most reasonable hypothesis indicated that there were four introgression events (Fig. 2F) and the reticulation events were all supported by QuIBL (Supplementary Data Table S6). For example, the ancient introgression could have occurred before the formation of the ancestor of Amaranthaceae, which may contribute to the alternative topologies of node I. This could also explain the discordant phenomena of nodes II and III identified by PhyloNet. Combining all the results, we speculate that there were ancient introgression events among the Caryophyllales. This may be the main factor contributing to the complexity of alternative topologies, and the phylogenetic discordance among the Caryophyllales.

### Karyotype evolution

From karyotype analyses (Fig. 3A), we found that for BTFR, which experienced the most complex whole-genome multiplication events, at least 114 chromosome fissions and 94 chromosome fusions were necessary to reach its current karyotype for each copy. *Portulaca amilis* needed at least 163 chromosome fissions and 175 chromosome fusion to reach its nine chromosomes. For *D. caryophyllus* and *G. paniculata*, at least 137 and 148 chromosome fissions and 143 and 147 chromosome fusion were necessary to reach their respective current karyotypes. Although the number of chromosomes of *G. paniculata* is greater than that of *D. caryophyllus*, there were more recombination events in *G. paniculata* than in *D. caryophyllus*. In *A. cruentus* at least 95 chromosome fissions and 99 chromosome fusions were necessary to reach its current karyotype. Among the species with no recently inferred WGDs, *Su. glauca*, *H. ammodendron*, *Sp. oleracea*, and *B. vulgaris*, at least 72, 55, 95, and 60 chromosome fissions and 83, 65, 108, and 70 chromosome fusions would have been needed to reach their current karyotypes, respectively. One thing to note is that *H. ammodendron* experienced fewest recombination events, and *P. amilis* experienced the most recombination events among the Caryophyllales.

### Evolution of pigment biosynthetic pathways and betalain content of different *Bougainvillea* cultivars

In the betalain biosynthesis pathway, BTFR contains the greatest number of genes (102), of which 19 were the result of a WGT event and one was the result of a γ event (Fig. 3B, Supplementary Data Table S8). The species most closely related to BTFR is *P. amilis*, which contains 91 betalain biosynthetic genes (Table 2, Fig. 3B). The lowest number of betalain biosynthetic genes was identified in *A. cruentus*, even fewer than in the anthocyanin-producing species (*G. paniculata*). We also found that fewer genes (13) in *P. amilis* belonged to either duplication, while others ranged from zero (*Bougainvillea*) to seven (*Sp. oleracea*) (Fig. 3B).

**Table 2:** Betalain biosynthetic pathway correlating gene numbers in different species.

	**CYP76AD**	**DODA**	**cDOPA5GT**	**Betanidin 5GT/6GT**	**Total** ^**+**^
*A. cruentus*	17	4	1	3	25
*B. vulgaris*	17	7	2	10	36
*Bougainvillea*	57	11	12	22	102
*P. amilis*	56	10	3	22	91
*H. ammodendron*	13	5	2	16	36
*Su. glauca*	14	6	1	18	39
*Sp. oleracea*	22	7	1	20	50
*G. paniculata* ^a^	8	2	1	18	29
*D. caryophyllus* ^a^	12	2	2	35	51

a Species producing anthocyanins. +, redundant total numbers.

In *D. caryophyllus* and *G. paniculata*, we detected 51 and 29 betalain biosynthetic related genes respectively, which is greater than the gene number in betalain-producing species such as *B. vulgaris* (36). We suspect that the expansion of glucosyltransferase (betanidin 5GT/6GT) could be the main factor contributing to anthocyanin biosynthesis [46]. Furthermore, the genes were duplicated through WGT and tandem duplication (Fig. 3), indicating the different evolutionary histories of the two closest species.

Regarding the anthocyanin biosynthesis pathway, betalain-producing species like *A. cruentus* and *H. ammodendron* were found to have lost the key genes (such as the *ANS* genes) in the pathway (Table 3, Fig. 3C). This is the most plausible reason why these species cannot produce anthocyanins. More *chalcone synthase* (*CHS*) genes were detected in *H. ammodendron* (21) and BTFR (37) compared with other Caryophyllales species that produce anthocyanin; this could be the main factor contributing to the greater number of anthocyanin biosynthetic genes. Previous research has identified *CHS* as a key enzyme in flavonoid biosynthesis [93]. Also, the expansion pathway of the *CHS* genes differed between the two species. For BTFR, this was caused by the recent WGD events (Fig. 3C, Supplementary Data Table S9), but for *H. ammodendron* the processes were proximal duplication, tandem duplication, and transposed duplication (Fig. 3C). Furthermore, there was an expansion of *flavanone 3-hydroxylase* (*F3H*) genes in *Bougainvillea*; despite the 10 genes generated by the recent WGD (Supplementary Data Table S9), there were 18 genes, which is a total greater than identified in any other species.

**Table 3:** Anthocyanin biosynthesis pathway correlating gene numbers in different species.

	***ANS***	***CHS***	***DFR***	***F3H***	***FLS***	***UFGT***	***4CL***	***CHI***	***PAL***	***C4H***	**Total** ^**+**^
*A. cruentus*	0	2	1	2	1	1	3	1	1	2	14
*B. vulgaris*	1	9	2	8	2	2	2	2	1	2	31
*Bougainvillea*	4	37	2	28	10	8	8	5	5	4	109
*P. amilis*	1	12	2	5	2	4	4	1	2	4	37
*H. ammodendron*	0	21	0	4	2	4	3	1	2	2	39
*Su. glauca*	2	8	3	11	1	1	3	1	1	2	33
*Sp. oleracea*	2	9	1	5	1	4	6	1	1	2	32
*G. paniculata* ^a^	1	9	4	6	2	4	4	2	4	3	39
*D. caryophyllus* ^a^	1	9	1	8	1	3	3	1	3	3	33

a Species producing anthocyanins. +, redundant total numbers.

**Figure 4:**
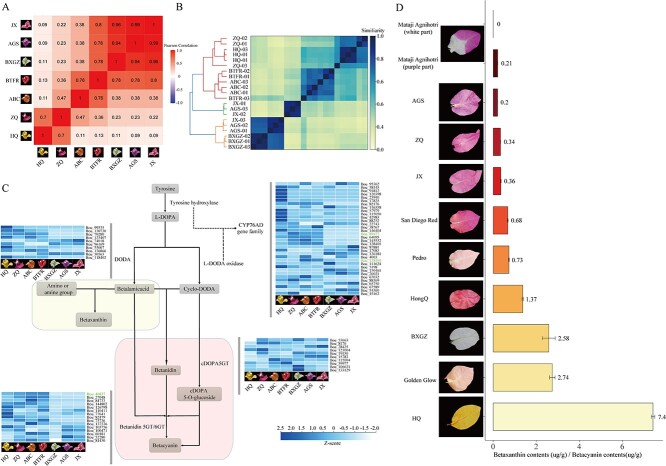
Transcriptome analysis and betalain contents in different cultivars. (A) Correlation heat map between FPKM values for genes taking part in the betalain biosynthesis pathway for different cultivars. (B) *K*-mer based distance tree. (C) Expression level of genes involved in betalain biosynthesis (average FPKM <1 values were removed and FPKM was normalized in each row). The gene ID marked in green indicates the shared DEGs among all the cultivars compared with BXGZ. (D) Ratio of betaxanthin to betacyanin content in different cultivars.

Further analysis was applied to seven *Bougainvillea* cultivars with different bract colors, from white to deep red and purple (Fig. 4A). From the PCA analysis variability between replicates was found to be minimal, and cultivars grouped into (i) *Bougainvillea* hybrid ‘Mrs Eva White’ (hereafter BXGZ), *Bougainvillea* hybrid ‘Rijnstar Pink’ (hereafter JX), and *Bougainvillea* hybrid ‘Elizabeth Angus’ (hereafter AGS), (ii) *Bougainvillea* hybrid ‘Sundance’ (hereafter ABC) and BTFR, and (iii) *Bougainvillea* hybrid ‘Firecracker Purple’ (hereafter ZQ) and *Bougainvillea* hybrid ‘Rijnstar Pink’ hybrid ‘Firecracker Yellow’ (hereafter HQ) (Supplementary Data Fig. S29).

Furthermore, the expression level of betalain biosynthetic genes was used to reveal the correlation between different cultivars, which confirmed the grouped results (Fig. 4A). The distance tree based on the *k*-mer value from transcriptome data which removed the low abundances still showed the same grouped results (Fig. 4B), demonstrating the stable expression patterns and relationships within the group. Through the cluster groups defined by different evidence, HQ and ZQ shared a similar expression pattern in the betalain biosynthetic pathway, but their bract color was divergent (yellow and magenta), while the group containing BXGZ, AGS, and JX exhibited further differences, although their expression pattern was more similar to each other (Fig. 4A and C, Supplementary Data Table S10).

We analyzed the differentially expressed genes (DEGs) between other cultivars and BXGZ (almost white). There were 44 genes with significantly different expression, and 9 genes were downregulated in ABC; 42 genes showed significantly different expression in BTFR, and among them 8 genes were downregulated; 49 and 41 DEGs were found in HQ and ZQ respectively, with 11 and 9 of these genes downregulated; both JX and AGS had 10 genes showing significantly different expression, and both had 3 genes showing downregulation (Supplementary Data Table S11). There were only three genes that showed significantly different expression among all the cultivars (Fig. 4C). Hence, most of the betalain pathway genes were upregulated compared with the light-colored BXGZ cultivar. Expression of betanidin 5GT/6GT genes was lower in BXGZ than in any other cultivar (Fig. 4C, Supplementary Data Table S10). Betanidin 5GT/6GT are the last enzymes that catalyze production of betalains. We infer that bract coloration requires high levels of expression through the entire betalain biosynthetic pathway, with high expression of betanidin 5GT/6GT genes being the key.

As for the expression of anthocyanin biosynthetic genes in *Bougainvillea*, we found that the *DFR* and *ANS* genes exhibited extremely low expression levels in every cultivar (Supplementary Data Table S12). We speculate that this may be one of the factors that contributes to the absence of anthocyanins in *Bougainvillea*.

*Dianthus caryophyllus* can produce anthocyanins but cannot produce betalains [6]. We selected transcriptome data from the same period (blooming stage) of petals from *D. caryophyllus* to analyze the expression patterns of the betalain biosynthetic pathway. We found that in *D. caryophyllus* the tyrosine decarboxylase (*DODA*) genes have very low levels of expression (Supplementary Data Table S13), which may hamper the biosynthesis of betalamic acid (Fig. 4C). This finding could explain the absence of betalains in this species even though this species contains the full betalain biosynthetic pathway.

Betalains were extracted from nine different *Bougainvillea* cultivars. The ratio of betaxanthin to betacyanin did not show a direct correlation with bract color (Fig. 4D, Supplementary Data Table S14). For example, *B. spectabilis* ‘Pedro’ (referred to as Pedro) exhibited a white color, close to that of *B. × buttiana* ‘Golden Glow’ (referred to as Golden Glow), they have totally different ratios of betaxanthin to betacyanin contents; and *B. spectabilis* ‘Tomato Red’ (referred to as HongQ), which exhibited a red/purple color, contained more betaxanthin than betacyanin. By extracting the betalains, we found that BXGZ and Mataji Agnihotri (white part), which exhibit light coloration, have lower levels of betaxanthin and betacyanin (~15 μg/g) than others, which range from 385 to 2575 μg/g (Supplementary Data Table S14). This indicates that total pigment content may directly affect bract color.

Correlation analysis between the betalain content and biosynthetic genes was performed (Supplementary Data Fig. S30). The results showed that there were 17 genes significantly correlated with the contents of betaxanthin and 1 gene significantly correlated with betacyanin. For betaxanthin, 16 of the significantly correlated genes showed a positive correlation and only 1 gene showed a negative correlation with the betaxanthin content. However, there was one gene that showed a negative correlation with betacyanin content.

## Discussion

### The *Bougainvillea × buttiana* ‘Mrs Butt’ genome and the evolutionary history among the nine Caryophyllales genomes

Based on state-of-the-art sequencing technology and assembly methods, we first generated a high-quality genome for *Bougainvillea* which we established to be ~5 Gb, with high-quality annotation. Despite the recent genome duplication events, the BTFR genome has the largest genome size, the explosion of the LTR expansion in a short period of time (~ 0.8 million years) may have contributed to the formation of its giga-genome. (Supplementary Data Table S2).

Here, we carefully analyzed the different whole-genome multiplication events among Caryophyllales (Fig. 2D) and demonstrated a diverse evolutionary history. Drawing on previous research [67, 76], we suggest that different whole-genome multiplication events have played an important role in the evolution of morphological and physiological diversity in Caryophyllales. Several regions followed the chromosome rearrangement events after the recent genome duplication in BTFR (Figs 1C and 2B). These regions may provide a great resource for further relative analysis, e.g. the gene flow between different cultivars [57]. To our knowledge, the life forms, genome size, and species richness are thought to cause different molecular evolution rates [68]; the higher mutation evolution rates usually correlate to a higher genome evolution rate [18]. We notice that *D. caryophyllus* and *G. paniculata* have the highest *μ* values, which represent the highest rate of diversification and the fastest evolutionary rate among the nine Caryophyllales genomes [7]. Both of these species produce sought-after ornamental flowers and have frequently been bred as hybrids. The lower *μ* rates in BTFR may be the result of its special cultivar background, as the original species.

Phylogenomic conflict, where gene trees conflict with species tree resolution, is common across genomes and throughout the Tree of Life [74, 75]. Genes with real and conflicting histories are expected within data sets due to biological processes like hybridization and ILS [58]. Since phylogenomic conflict often represents the imprint of past population genetic processes on the genome, studying its correlation with other macroevolutionary patterns may shed light on the microevolutionary processes underlying major transitions across the Tree of Life [66]. Our results showed discordance between gene trees and species tree among different types of datasets. Although the supermatrix species tree has already resolved the relationships among the nine species in Caryophyllales, we identified multiple instances of strongly supported conflicts in some nodes through different datasets (Fig. 2E, Supplementary Data Fig. S27), and further detected that most of the incongruences can be explained by introgression (Supplementary Data Tables S6 and S7). Although we did not find cytonuclear discordance, ancient hybridization could exist because chloroplast capture does not always accompany introgression [80]. Our results suggest that ancestors of different species may experience complex hybridization events, a scenario similar to that suggested by previous research [4, 45].

### Evolutionary history of pigment biosynthesis and expression patterns of bract color

Based on the analysis results, the pigment biosynthetic pathways of different species experienced different expansion events, and the duplication types during the expansion history were varied. This reveals that different species have their own duplication patterns and evolutionary history of the pigment biosynthetic pathways. Fewer genes were retained from the WGD in the pigment pathway, as discussed in prior research [69]. The whole-genome multiplication events were often followed by diploidization and duplicated genes have been lost over a few million years [55]. Hence, fewer genes are thought to be retained from the whole-genome multiplication, much less the ancient γ (Supplementary Data Tables S8 and S9). Proximal duplication and tandem duplication could be the main duplication types in two pigment biosynthesis pathways among different species (Fig. 3B and C). The proximal duplication may originate from the ancient tandem duplications that have been interrupted by the other genes or from localized transposon activities [28]. Tandem duplications were closely sited near each other in the same chromosome and several research studies have identified that the gene neighborhood tends to be co-regulated [92]. Tandem genes could help the plant to adapt to rapidly changing environments [38].

A high diversity of the betalain biosynthetic pathway was revealed through the transcriptome analysis of different cultivars in *Bougainvillea*. For example, the group containing ZQ and HQ, which were thought to be closely related germplasms sharing almost the same expression pattern in the betalain biosynthetic pathway, had different bract colors. Although the expression pattern does not show a positive correlation with bract color, the DEG analysis demonstrated that higher expression and more genes in the pathway are needed to generate the bract colors (Fig. 4C, Supplementary Data Table S11).

Overall, we provided the BTFR genome and used multiple genomes to reveal that introgression among Caryophyllales was the main factor that contributed to the discordance between species tree and gene trees. The complex whole-genome multiplication events and karyotype evolutionary results indicated that the nine Caryophyllales species had their own individual evolutionary pattern. Furthermore, the pigment biosynthetic pathways in each genome exhibited a species-specific evolutionary history, and did not correlate with the genomic evolutionary process. More generally, the analysis of cultivars with different colors provided new insight into the relationship between bract color and gene expression.

## Materials and methods

### Materials and sequencing

Young green leaves (third to fourth internode) of BTFR were collected and stored immediately at −80°C for genome sequencing, and the genomic DNA was prepared by the CTAB extraction method, followed by purification with a Qiagen extraction kit (catalog no. 13343) using the manufacturer’s instructions. A SMRTbell target size library was constructed for sequencing using either 10- or 20-kb preparation solutions. Sequencing was performed on a PacBio Sequel II instrument with Sequencing Primer V2 and a Sequel II Binding Kit 2.0 in Grandomics. HiFi data were obtained using the CCS algorithm (v.6.0.0). Young roots of BTFR shorter than 2 cm were used to perform karyotype analysis and confirm the chromosome number.

For transcriptome samples, bracts from BTFR, ZQ, HQ, ABC, AGS, BXGZ, and JX at the stage when the second bud opens wider at the top and inner surface of the perianth is visible were sampled (Fig. 2B). All samples included three biological replicates and were collected from at least three plants (six three-flowered umbels per plant).

The *Bougainvillea* cultivars of *B. peruviana* ‘Mary Palmer’ (referred to as Mataji Agnihotri), Pedro, *B. × buttiana* ‘San Diego Red’ (referred to as San Diego Red), HongQ, Golden Glow, AGS, ZQ, JX, BXGZ, and HQ from different sampling dates were used to extract the betaxanthin and betacyanin contents from the bracts as described previously [91].

### Genome assembly and annotation

A genome survey was performed using FindGSE [81] (*k*-mer = 31). Hifiasm v.0.16.1-r375 [15] was used to generate the assembly contigs with the HiFi reads. The scaffolds were sorted and assembled onto chromosomes using JUICER [22] and 3D-DNA [21]. BUSCO v.5.2.2 [60] was used to evaluate the quality of the assembled genome by using the database ‘eudicots_odb10’. The CC ratio [88] and KAT (K-mer Analysis Toolkit) [61] were also used to evaluate the quality of the assembled genome.

The genome was masked by RepeatMasker and RepeatModeler [27]. A combined strategy based on homology evidence, *de novo* prediction, and EST (expressed sequence tag) evidence was used to predict the gene structure. These proteins were aligned to the repeat-masked genome by Exonerate (v.2.4.0). For *de novo* gene prediction, we used the BRAKER v.2.1.6 [40] pipeline, which combined GeneMark-ET v.4.68_lic [79] and Augustus v.3.4.0 [78]. The RNA-seq reads, which were quality-controlled by FastQC [2], were assembled using Trinity v.2.8.5 [36], after being cleaned by SeqClean [14], then mapped to the repeat-masked genome by minimap2 [52], and the gene structure was predicted by PASA v.2.5.0 [35] as EST evidence. Finally, EvidenceModeler [37] was used to generate a non-redundant gene set. The non-redundant gene set was further filtered by gFACs [9] to remove the incompletes genes.

Gene functions were assigned according to the best match by aligning the protein sequences to the Swiss-Prot and NCBI non-redundant (NR) [72] databases using Blastp (E-value = 1e−5) [11]. The motif and domains were annotated by Interproscan (v.5.52–86.0) [44] and PfamScan (v.3.3.2) [62].

EDTA v.2.1.0 [65] was used to fully annotate the transposable elements after completing genome structure prediction.

### Whole-genome duplication and karyotype analysis

The toolkit WGDI [82] was used to infer polyploidization events in nine different Caryophyllales species. Collinear genes were first identified with the parameter ‘-icl’ of WGDI within each genome, and collinear gene dot plots were used to count the syntenic ratios between different species to confirm the polyploidy level of each species. The frequencies of *K*_s_ values between collinear genes were estimated using the Nei–Gojobori approach. The median *K*_s_ values of each block were selected to perform *K*_s_ peak fitting using WGDI with the parameter ‘-pf’. DupGen_Finder [69] with default parameters was used to distinguish genes remaining after different duplication events, and setting *V. vinifera* as outgroup.

The ancestor of core eudicots karyotype (ACEK) information was obtained from the WGDI. We used this ACEK information because it recovered many previous blank regions compared with previous karyotype results. We used ‘-akr’ to update the ACEK information by adding the genetic information from the *B. vulgaris* [19], *Sp. oleracea* [10], *A. cruentus* [56], *Su. glauca* [96], *Haloxylon ammodendron* [87], *P. amilis* [31], *D. caryophyllus* [100], *G. paniculata* [51], and *Bougainvillea*.

### Phylogenomic analysis

The longest transcripts of each gene from *B. vulgaris*, *A. cruentus*, *Su. glauca*, *Sp. oleracea*, *H. ammodendron*, *P. amilis*, *D. caryophyllus*, *G. paniculata*, *V. vinifera* [25], and BTFR were used to perform the phylogenomic analysis. Orthofinder v.2.5.4 [24] was used to cluster genes into different groups. Groups were divided into four datasets, the SCGs, SSC genes, LC2 genes, and LC5 genes. The SCGs were generated by using the single duplicated genome (ChrA) of BTFR as input for the OrthoFinder analysis and generating the single-copy gene groups. The SSC groups were generated by OrthoFinder, of which BTFR contains two copies and others contain only one copy. The LC2 group includes every species that contains genes with two or fewer copies. The LC5 group is every species that contain genes with five or fewer copies. MAFFT [48] was used to process multiple sequence alignments (MSAs), and trimal [12] was adopted for trimming the MSA results. IQTREE v.2.2.0.3 [64] was used to perform the phylogenetic analysis at the single-gene and concatenated species tree levels. TreeShrink [59] was selected to reduce the influence of long branch attraction in the single-gene trees. ASTRAL [98] was used to infer the coalescent species tree. The calibration time of divergence was obtained from TimeTree [49] as the benchmark for the following analysis. We used mcmctree [94] to calculate divergence time based on the species trees generated by IQTREE. The resulting trace files were inspected using Tracer v.1.7 [70].

The gene trees that were used for the further analysis were filtered by the following steps: (i) removal of the gene trees that may be influenced by long branch attraction; (ii) removal of trees that contained the branch length ‘0’; (iii) removal of topologies that were different from the species tree generated from IQTEE and ASTRAL. We ran QuIBL [23] on every triplet individually under default parameters with the number of steps set to 50 and setting *V. vinifera* as the outgroup.

We also inferred the phylogenetic networks using PhyloNet [89], via the ‘InferNetwork_MPL’ command. One to five reticulations were set to infer the phylogenetic networks. The result was visualized by Dendroscope [42]. The *k*-mer-based distance tree was built using Sourmash [8].

### Pathway gene identification

Protein sets were compared with the pathway genes by Blastp (positive hits with at least 50% amino acid identity and E-value of 1e−5). The positive hit proteins were further tested if they contained the specific domain identified by HMMER [63].

DEG analysis was performed by DEseq2 [54]. We calculated the expression level of each transcript using the fragments per kilobase of exon per million mapped reads (FPKM) method. The standard of the absolute log_2_-transformed fold-change values >2 with a *q*-value of 0.05. The correlation analysis between betalain content and FPKM was performed using ComplexHeatmap [33].

## Supplementary Material

Web_Material_uhad124

## Data Availability

The genome assembly sequences, gene annotations and transcriptome data are publicly available in the China National GeneBank (https://www.cngb.org/) under project number CNP0004115.
