# Correction to: A high-quality *Bougainvillea* genome provides new insights into evolutionary history and pigment biosynthetic pathways in the Caryophyllales

**DOI:** 10.1093/hr/uhad264

**Published:** 2023-06-13

This is a correction to: Lan Lan, Huiqi Zhao, Suxia Xu, Shenglong Kan, Xiaoni Zhang, Weichao Liu, Xuezhu Liao, Luke R Tembrock, Yonglin Ren, Wayne Reeve, Jun Yang, Zhiqiang Wu, A high-quality *Bougainvillea* genome provides new insights into evolutionary history and pigment biosynthetic pathways in the Caryophyllales, *Horticulture Research*, Volume 10, Issue 8, August 2023, uhad124, https://doi.org/10.1093/hr/uhad124

In the originally published version of this manuscript, the conclusion in the paper was incorrect: there should be two WGD events and one WGT (γ) event in Bougainvillea genome, not the conclusion in the paper of one WGD event and two WGT (γ) events.

This false conclusion was caused by the misdirection of the recent WGD events, which doubled the syntenic depth for the WGD and made it look like a WGT event.

The author apologize for the wrong conclusion and have corrected Figure 2 and Figure 3. Several descriptions in the main text have also been corrected accordingly.

**Figure 2:**
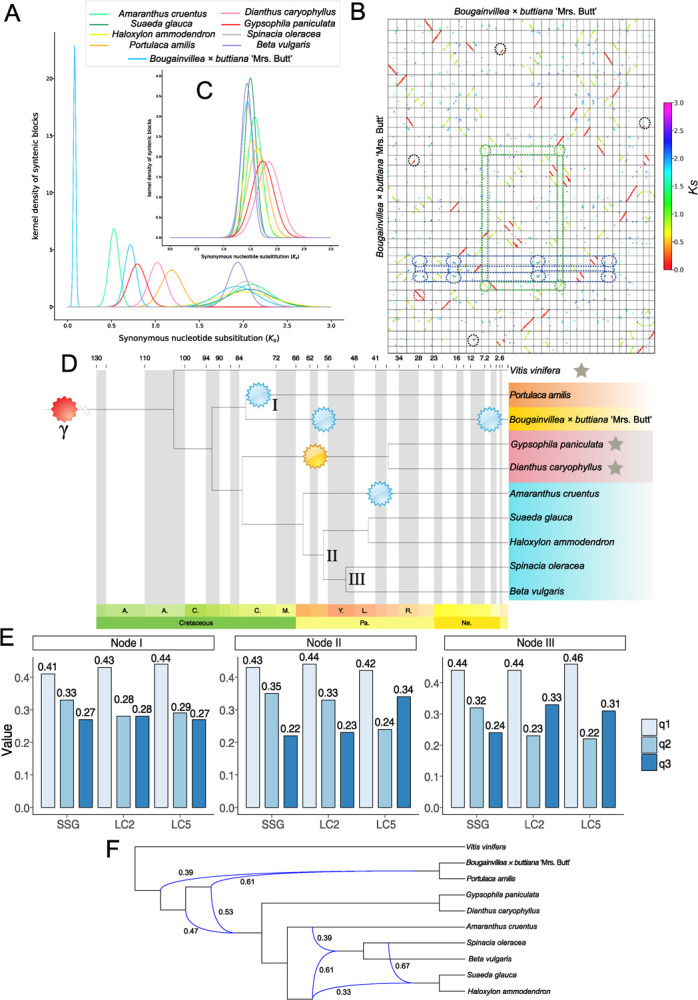


**Figure 3:**
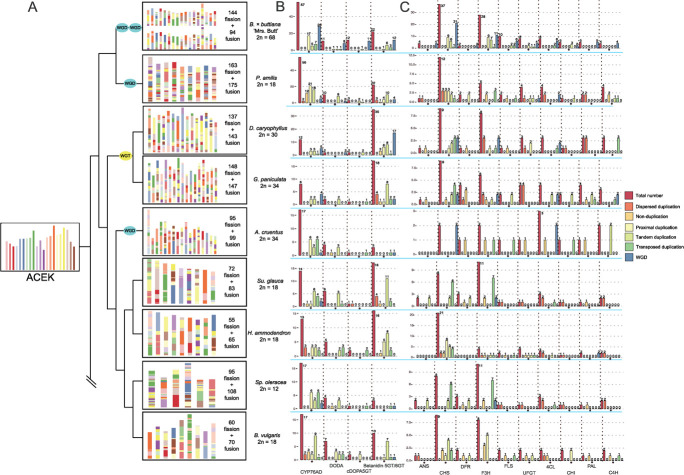


This error has been corrected online.

