# Supplementary material for: A high-quality Bougainvillea genome provides new insights into evolutionary history and pigment biosynthetic pathways in the Caryophyllales
Source: Hortic Res. 2023 Jun 13;10(8):uhad124. doi: 10.1093/hr/uhad124 (PMC10405137; doi:10.1093/hr/uhad124)
Supplement: Web_Material_uhad124 [file web_material_uhad124.zip › Supplymenttary_figures.pdf]

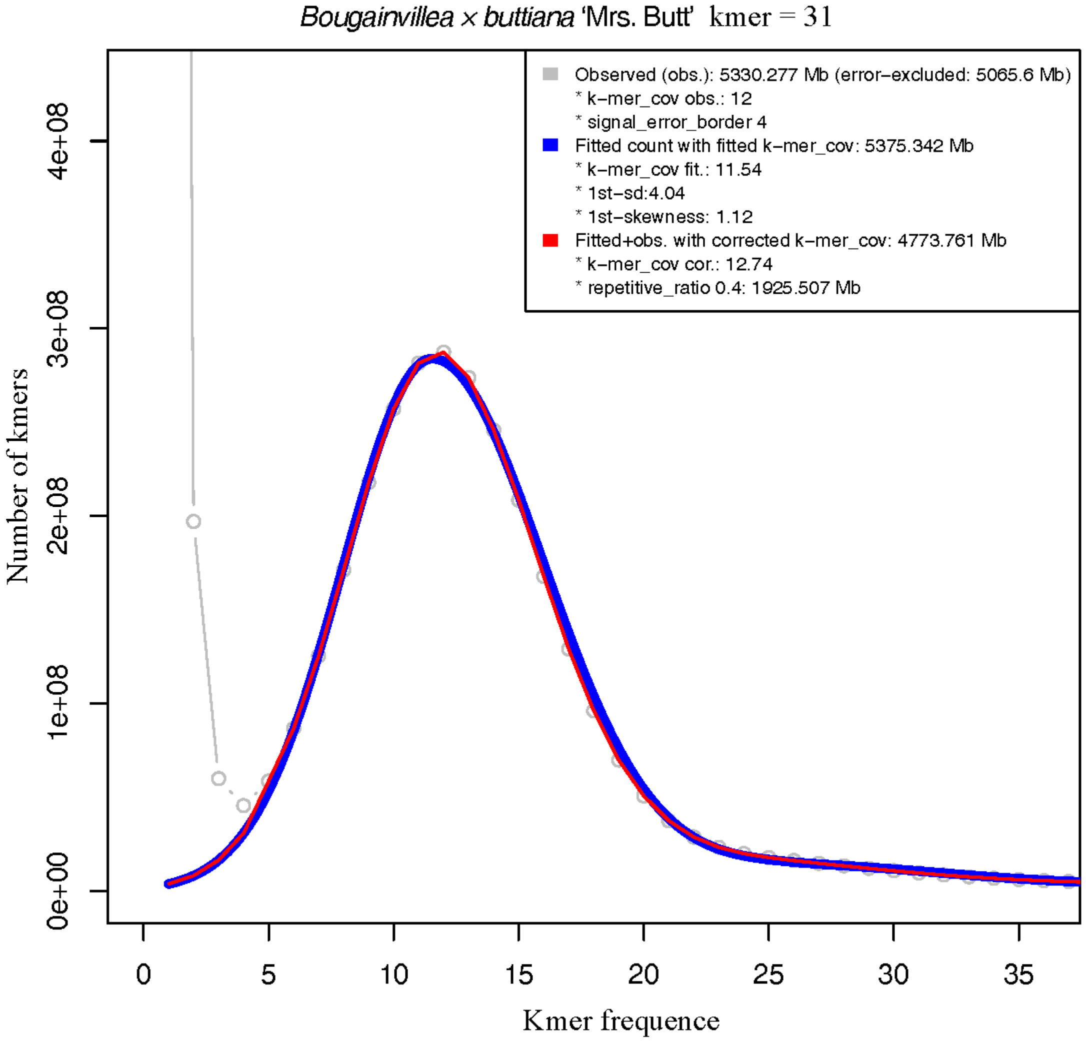

Figure S1. The genome survey result of *B.* × *buttiana* 'Mrs. Butt'.

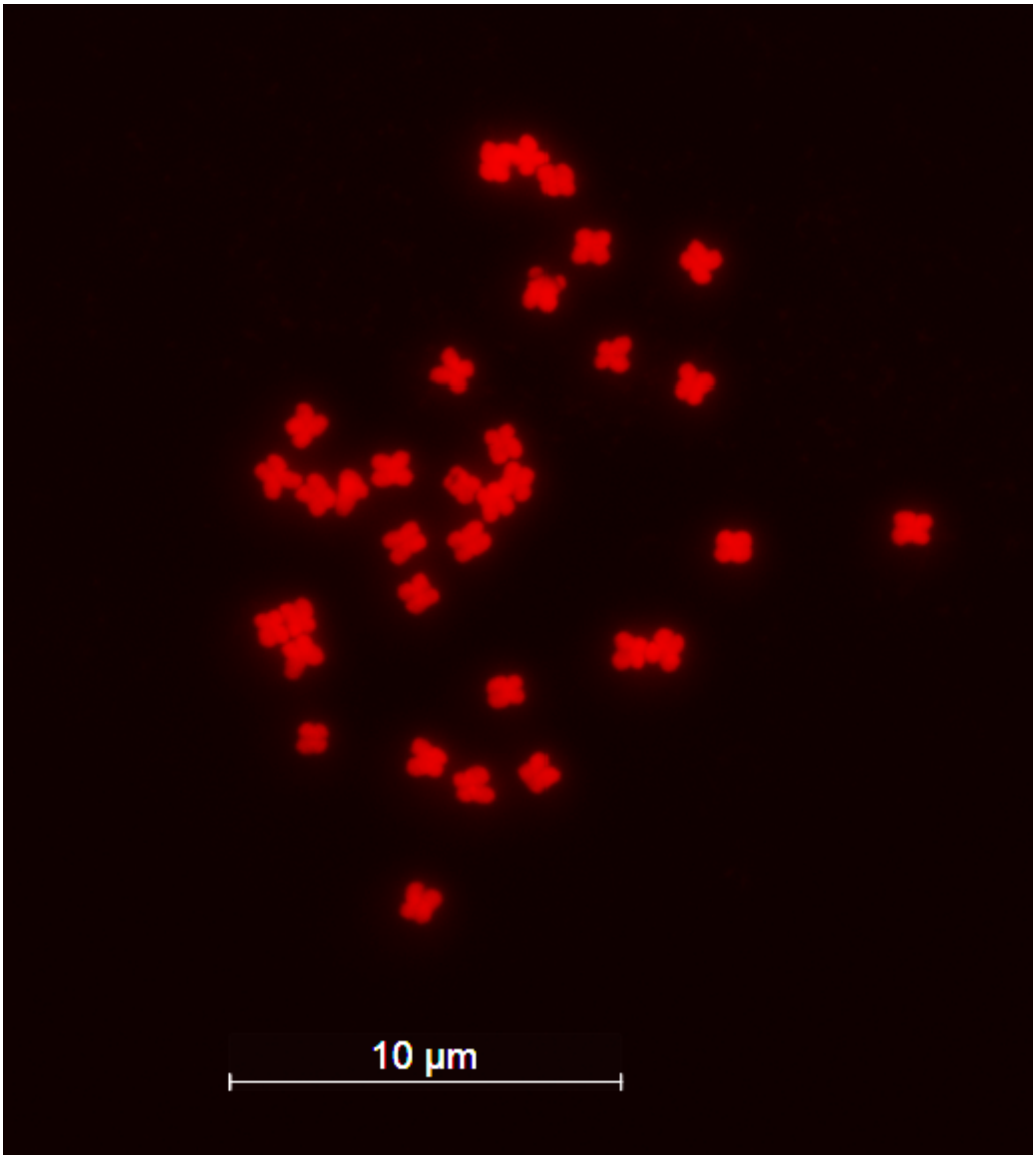

Figure S2. The karyotype of *B.* × *buttiana* ‘Mrs. Butt’.

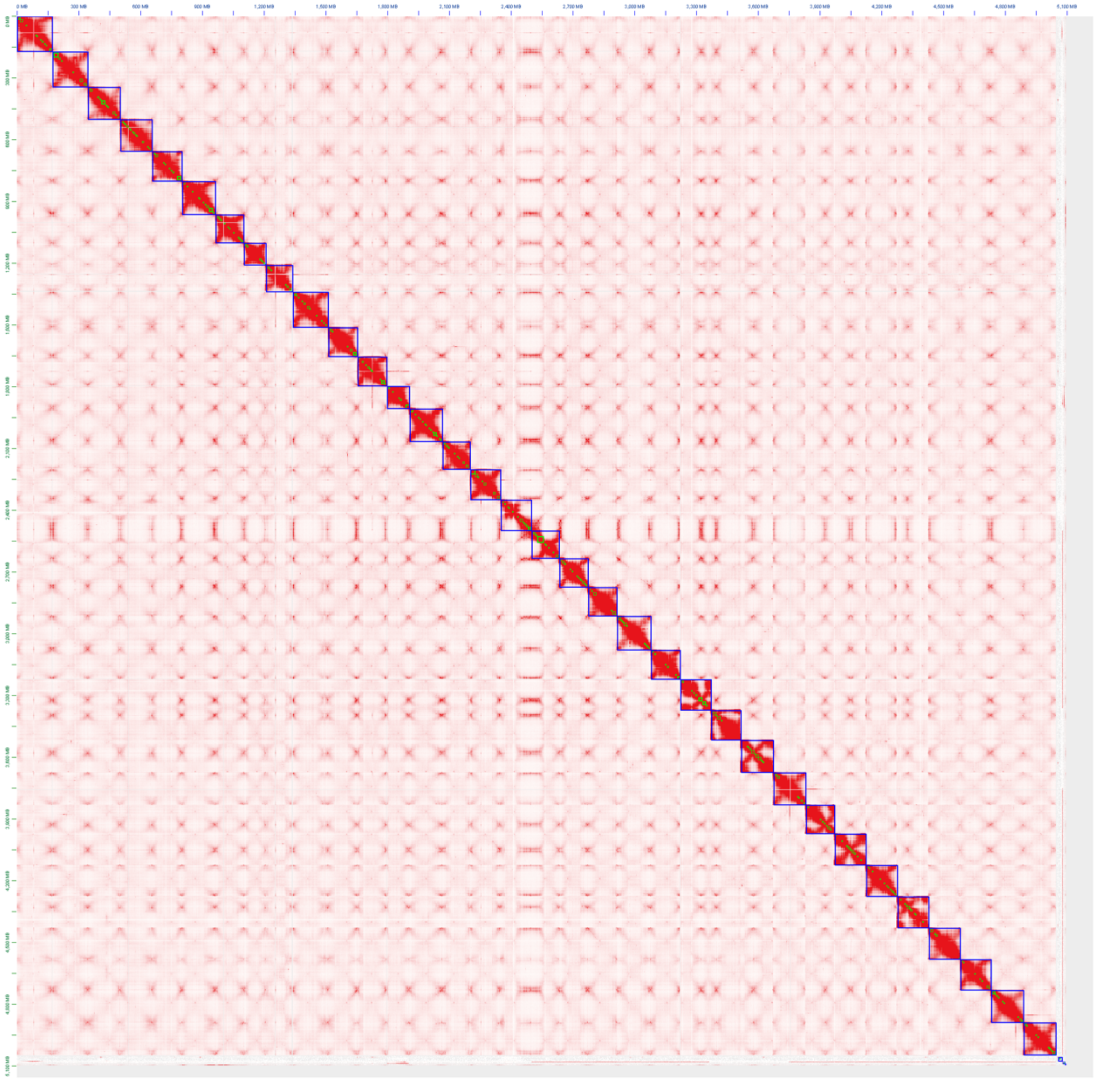

Figure S3. *B.* × *buttiana* ‘Mrs. Butt’genome-wide all-by-all HiC-interaction heat map.

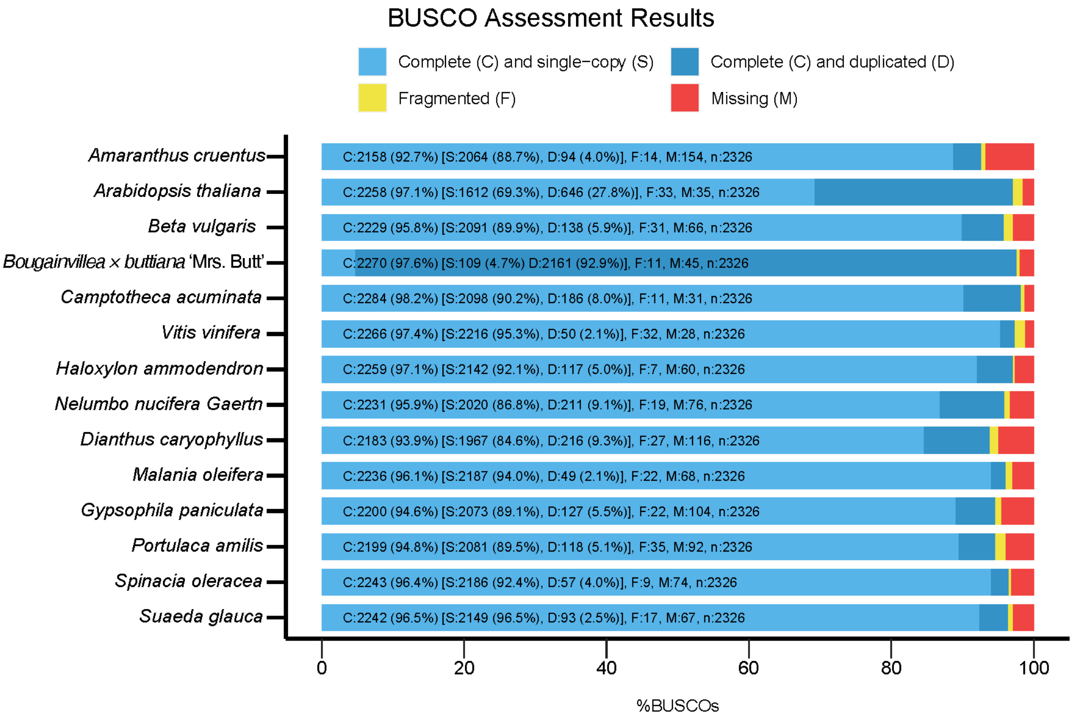

Figure S4. The BUSCO evaluation results among the 9 Caryophyllales genomes.

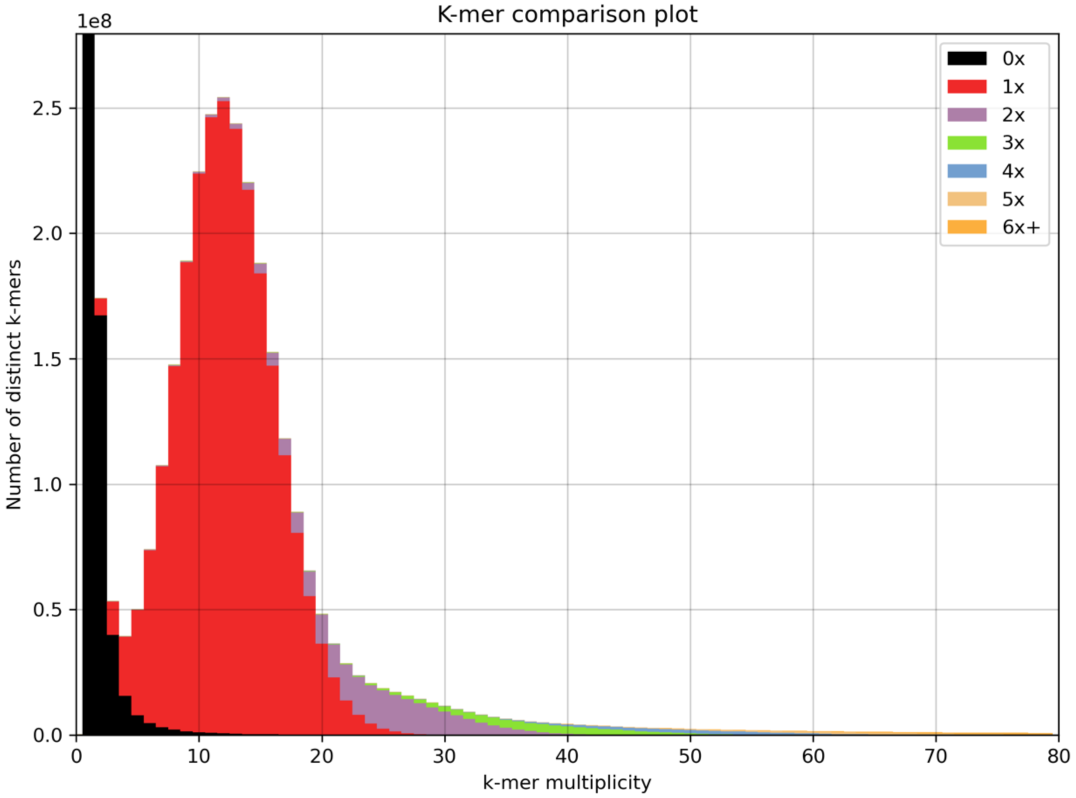

Figure S5. The KAT evaluation kmer spectrum plot of *B.* × *buttiana* 'Mrs. Butt' genome.

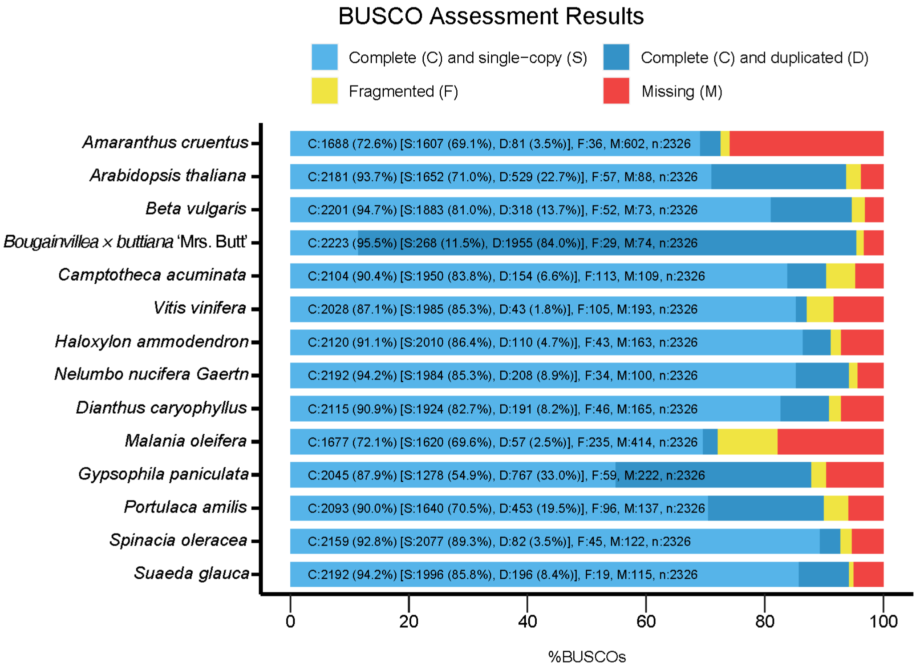

Figure S6. The BUSCO evaluation results among the 9 Caryophyllales genomes' annotation results.

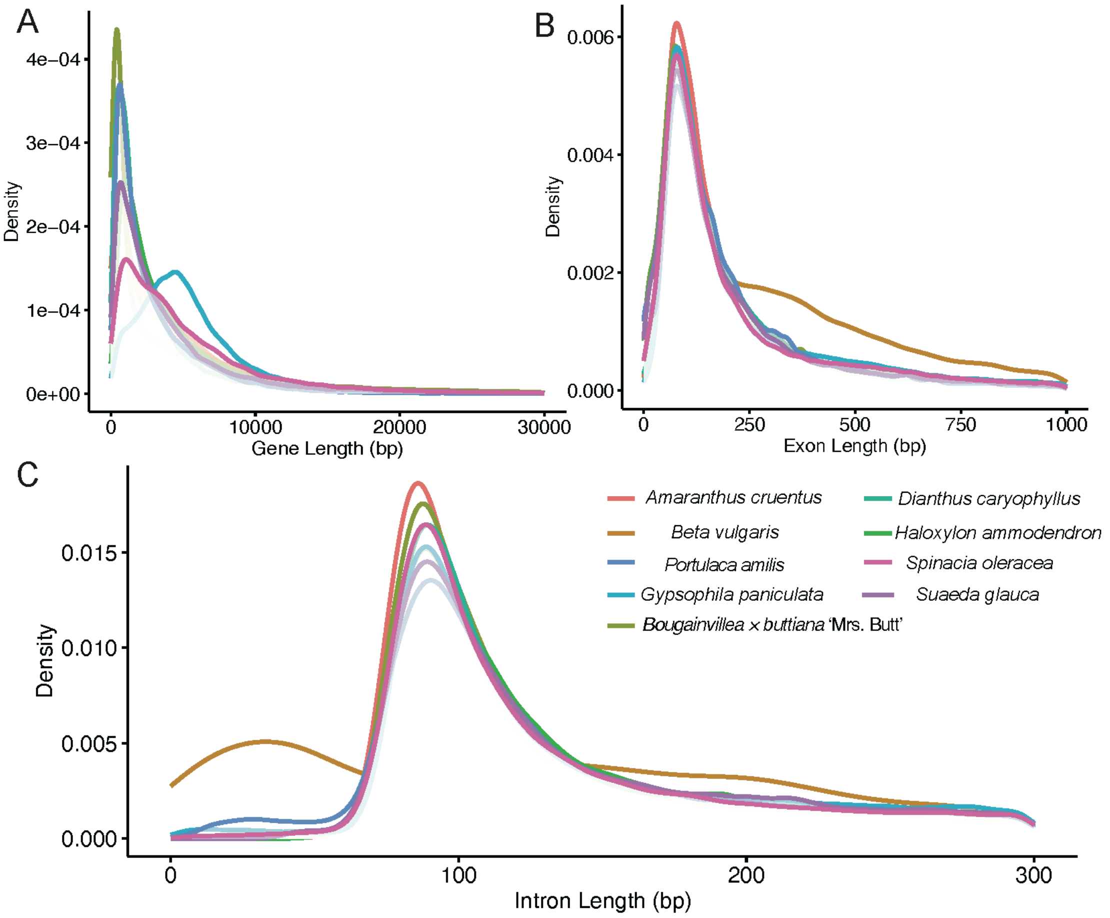

Figure S7. The distribution of gene (A), exon (B) and intron (C) length distribution.

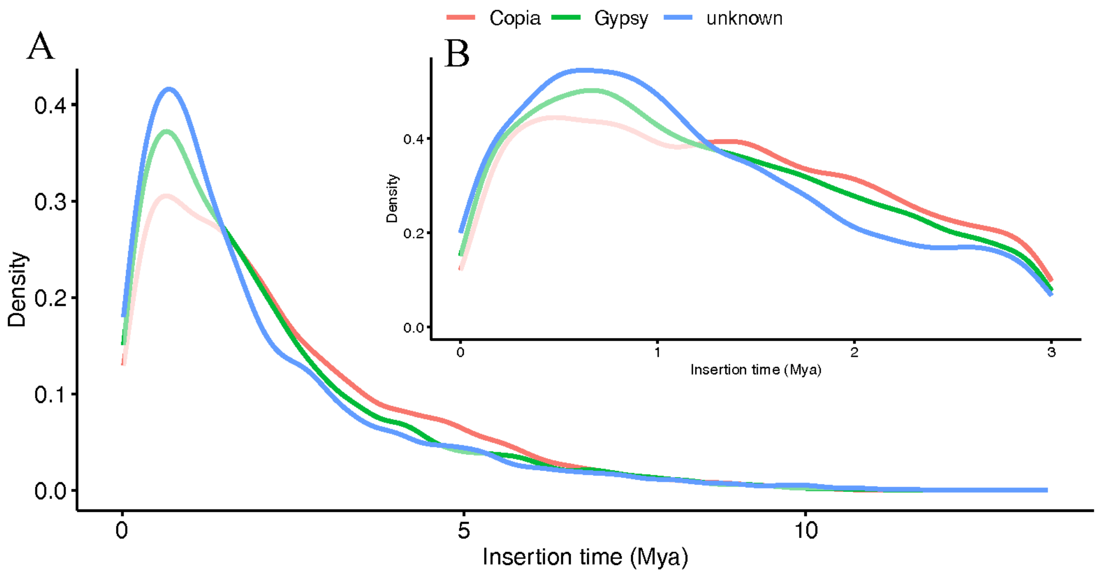

Figure S8. The LTR insertion time in *B.* × *buttiana* ‘Mrs. Butt’ genome. (A) The total distribution insertion time of different LTR; (B) The part of the plot in insertion time from 0~3Mya of (A).

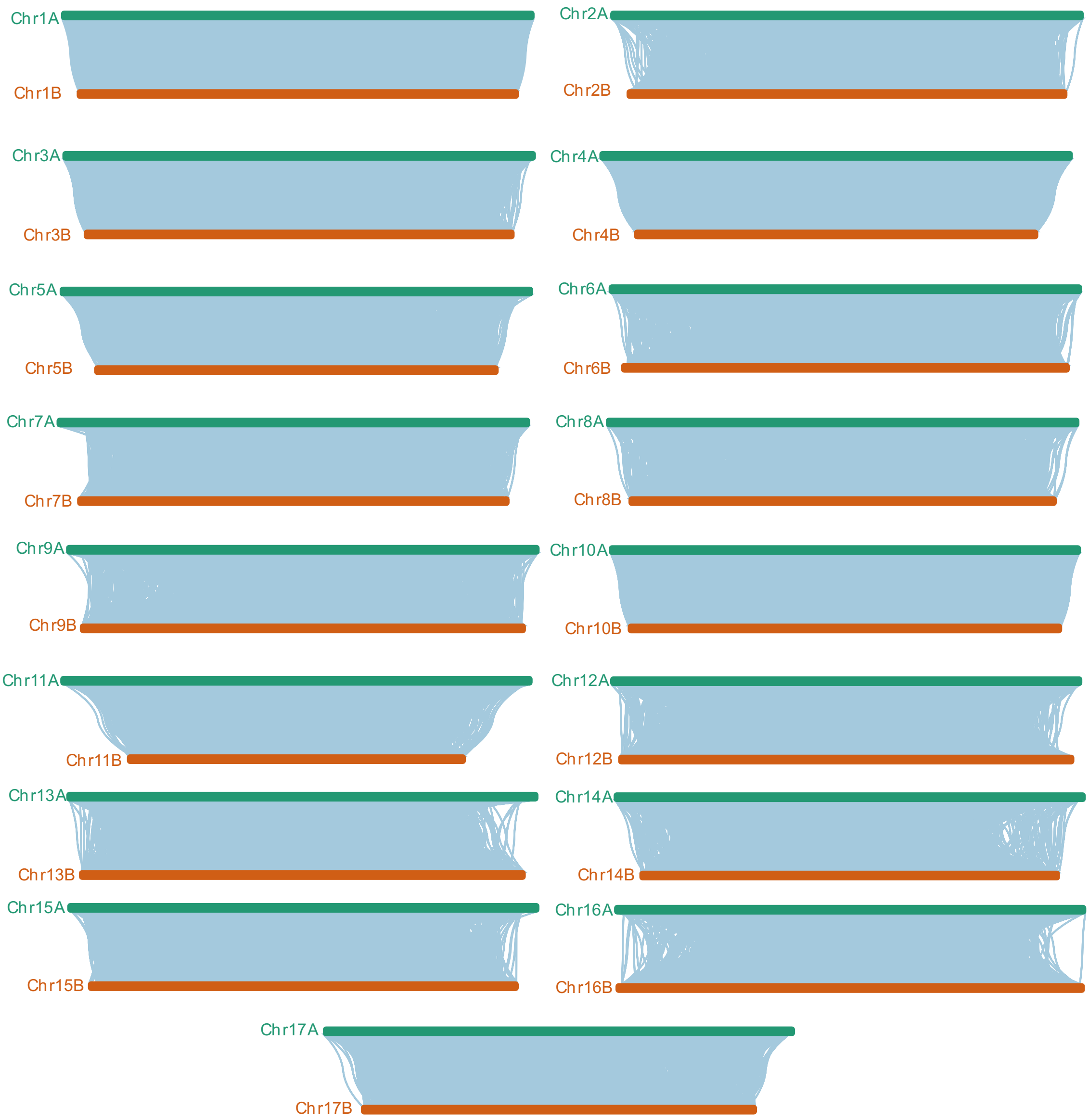

Figure S9. The co-linearity analysis results based on the BTFR genome data which experienced the recent WGD events. The A and B means the homochromosome generated by the WGD events.

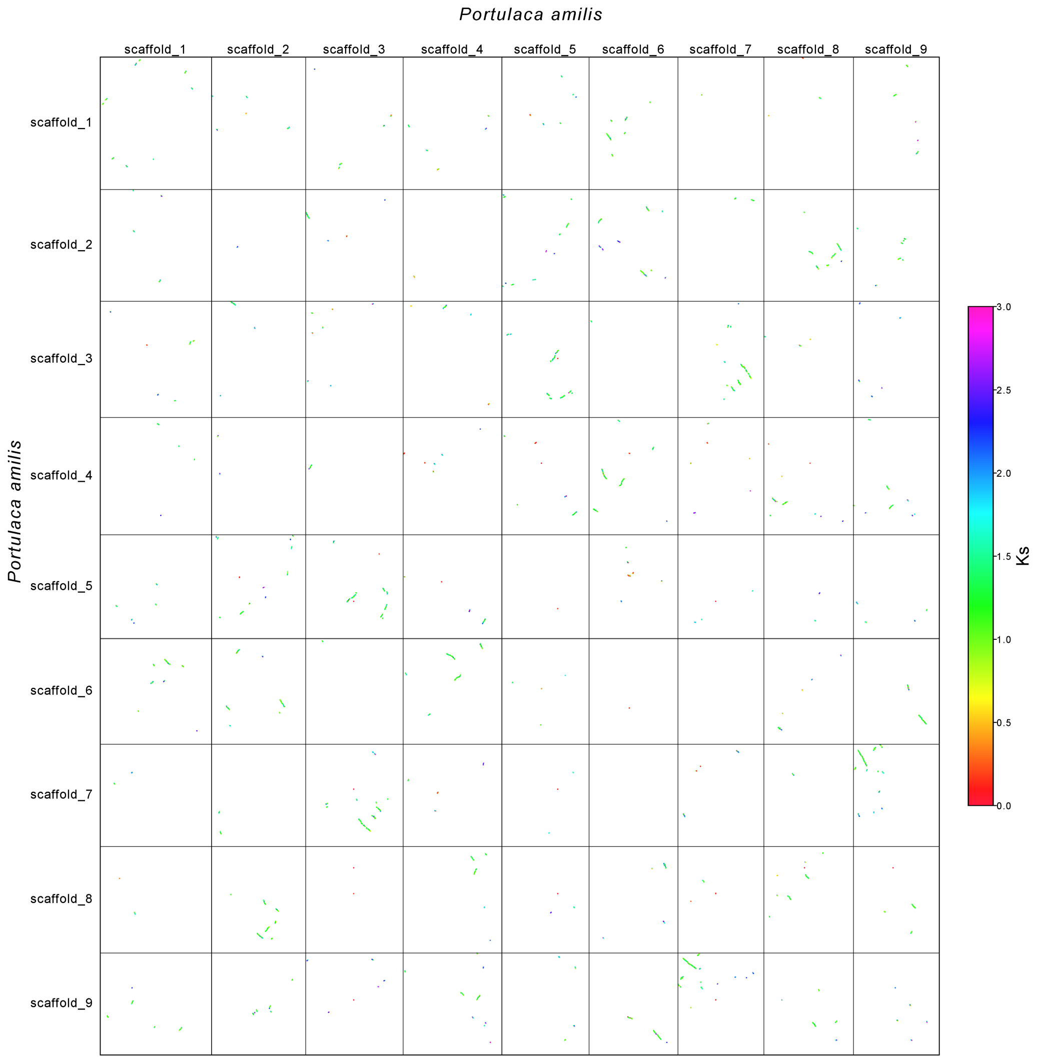

Figure S10. The synteny blocks of the *P. amilis* genome.

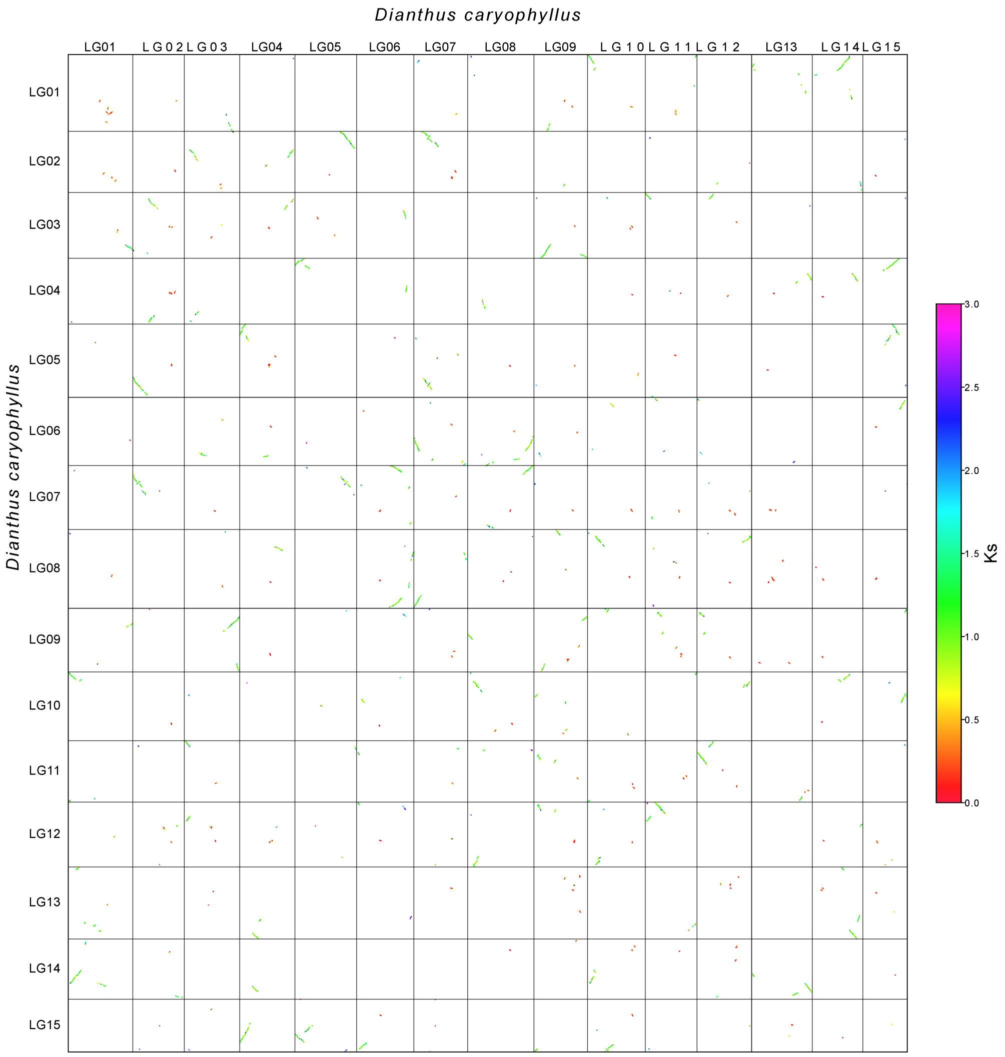

Figure S11. The synteny blocks of the *D. caryophyllus* genome.

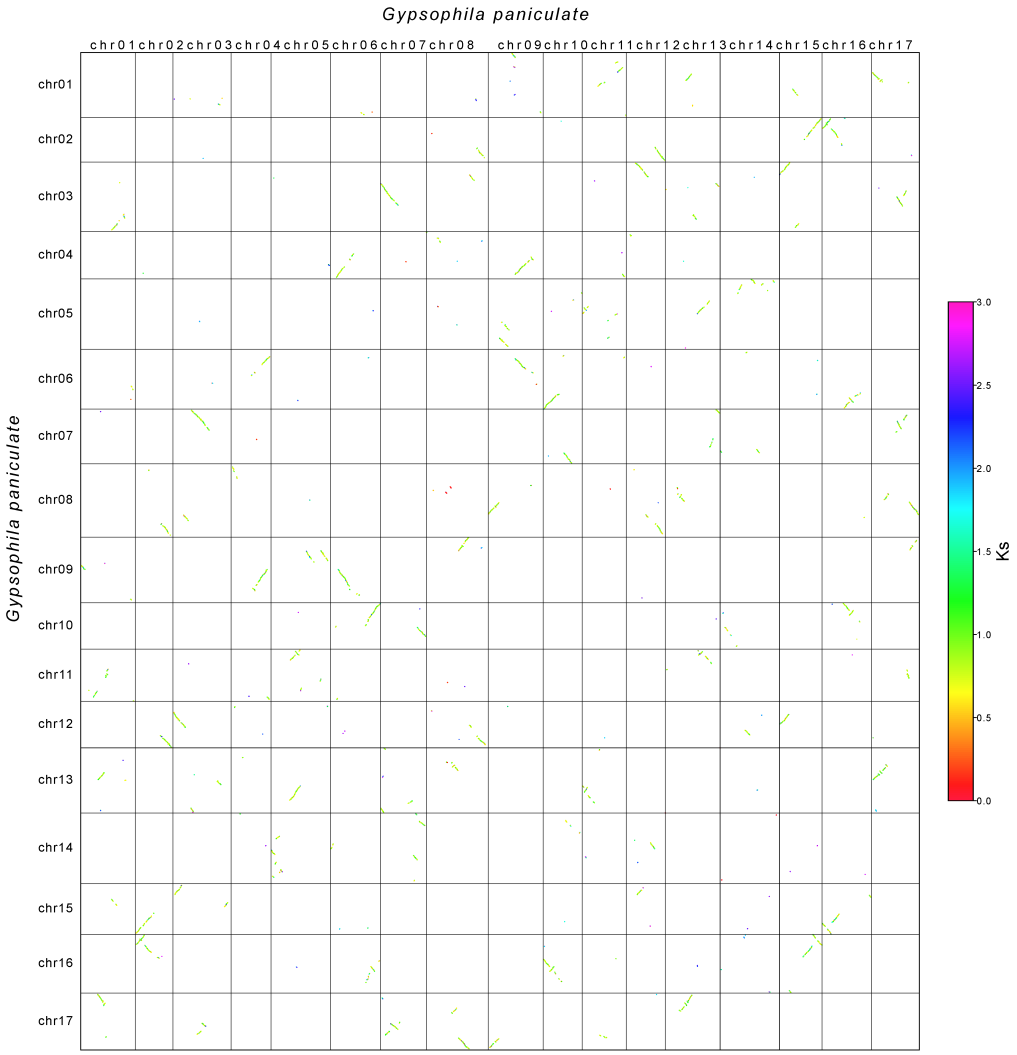

Figure S12. The synteny blocks of the *G. paniculate* genome.

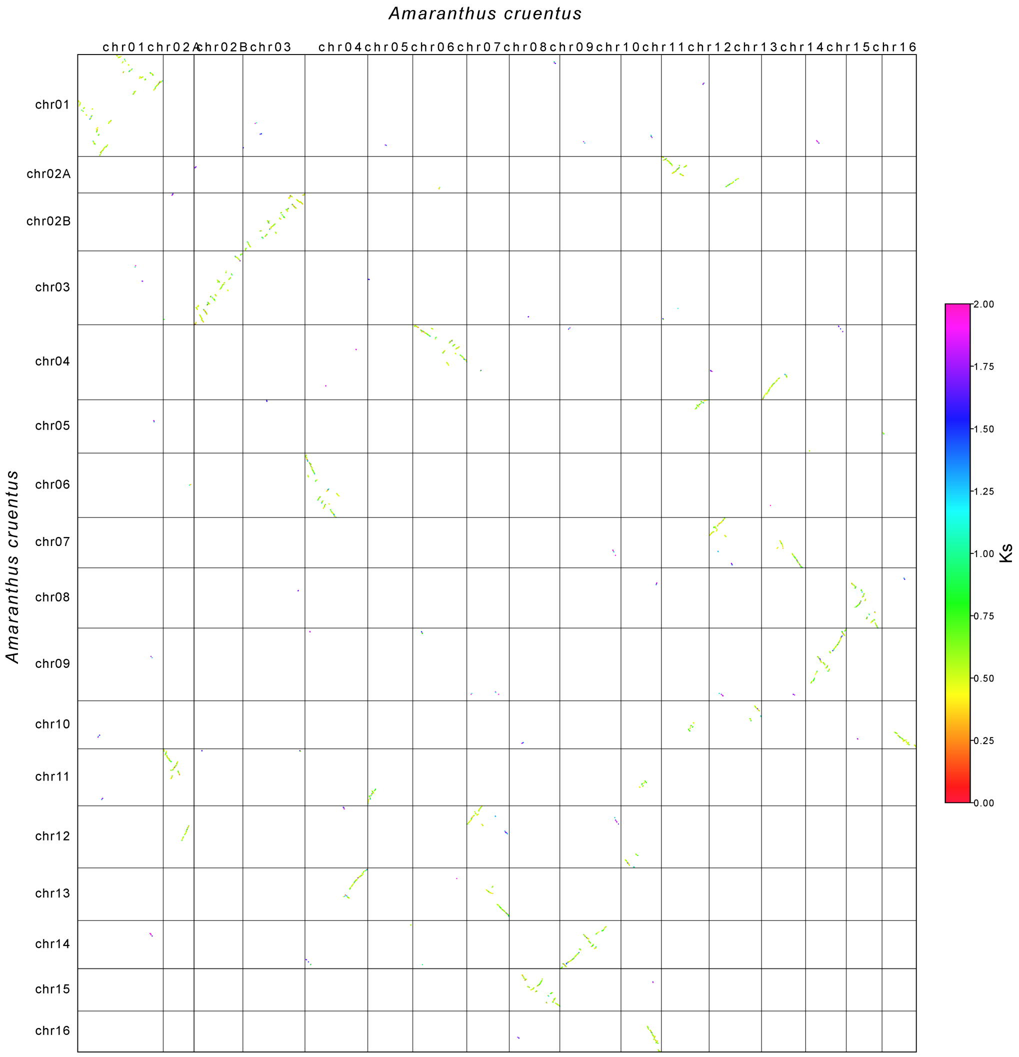

Figure S13. The synteny blocks of the *A. cruentus* genome.

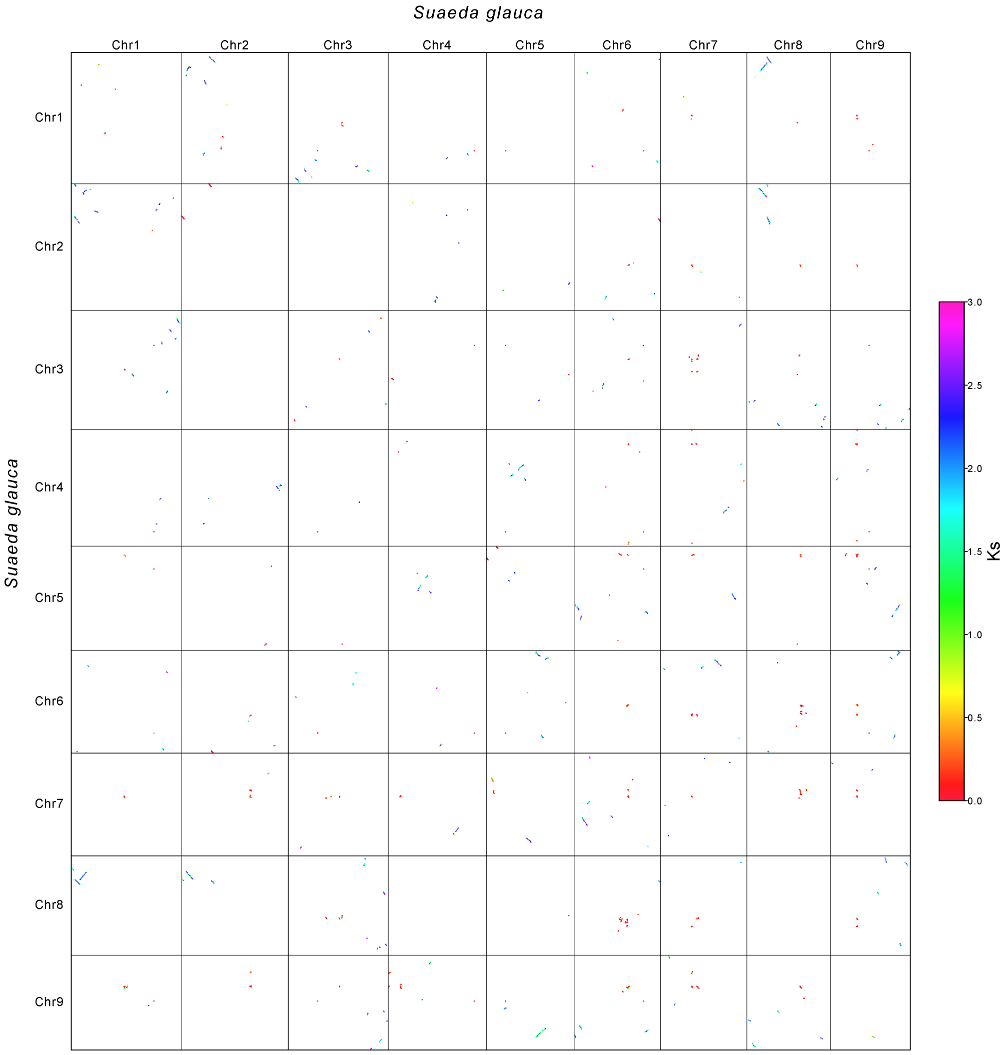

Figure S14. The synteny blocks of the *S. glauca* genome.

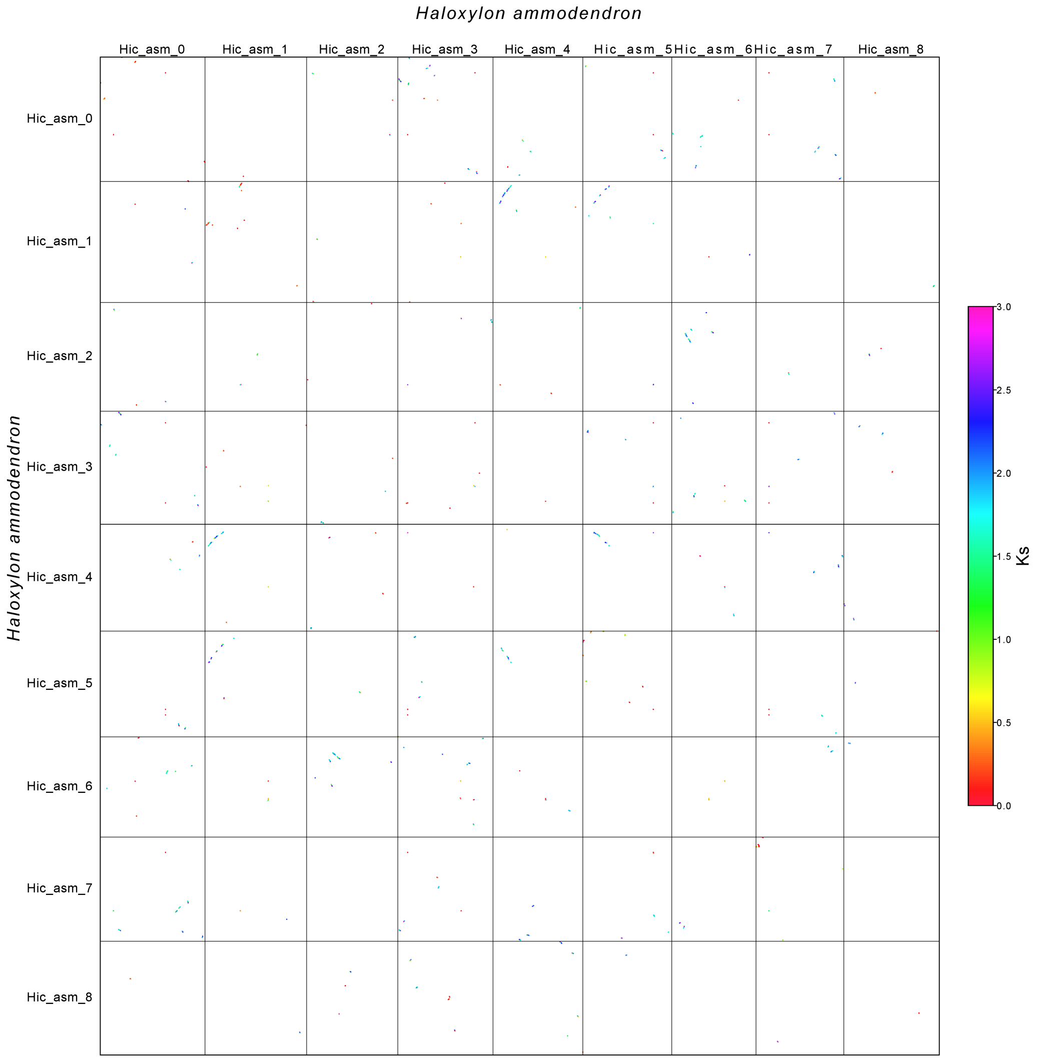

Figure S15. The synteny blocks of the *H. ammodendron* genome.

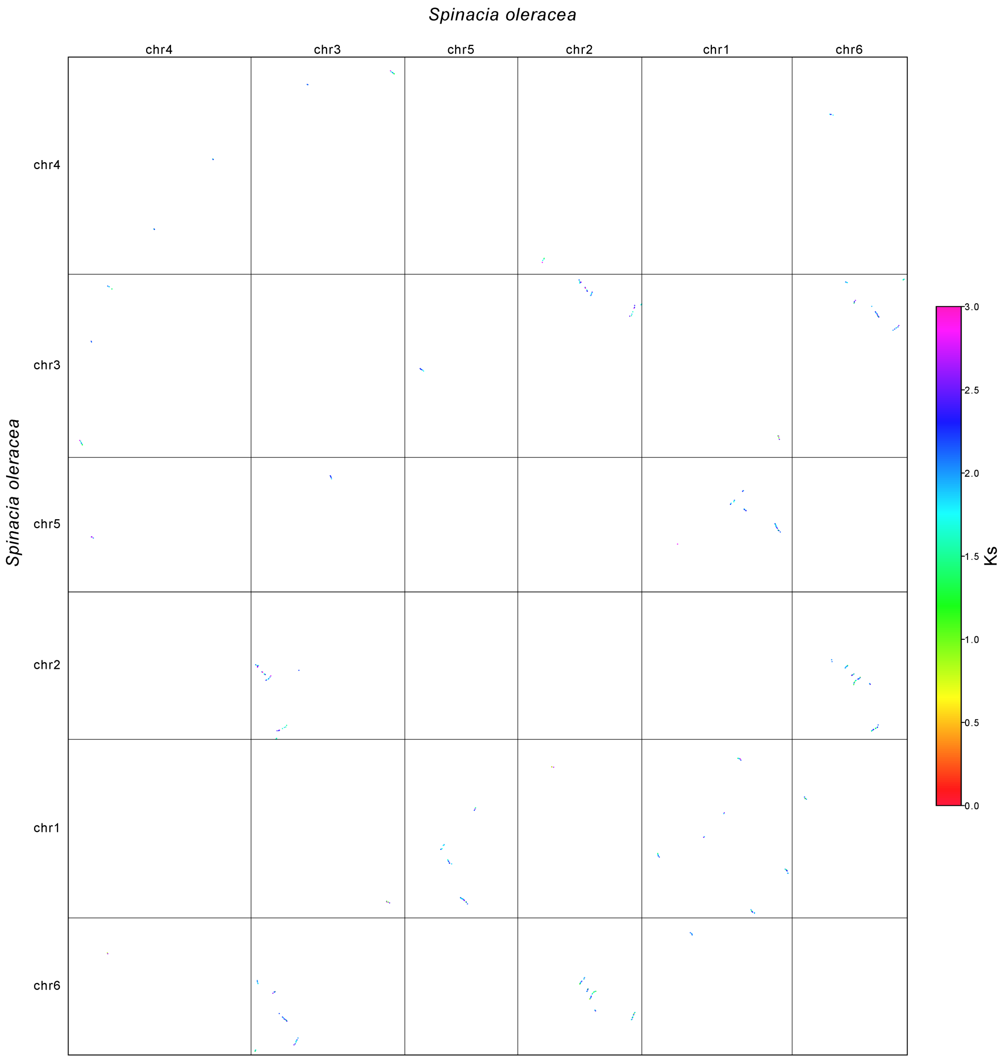

Figure S16. The synteny blocks of the *S. oleracea* genome.

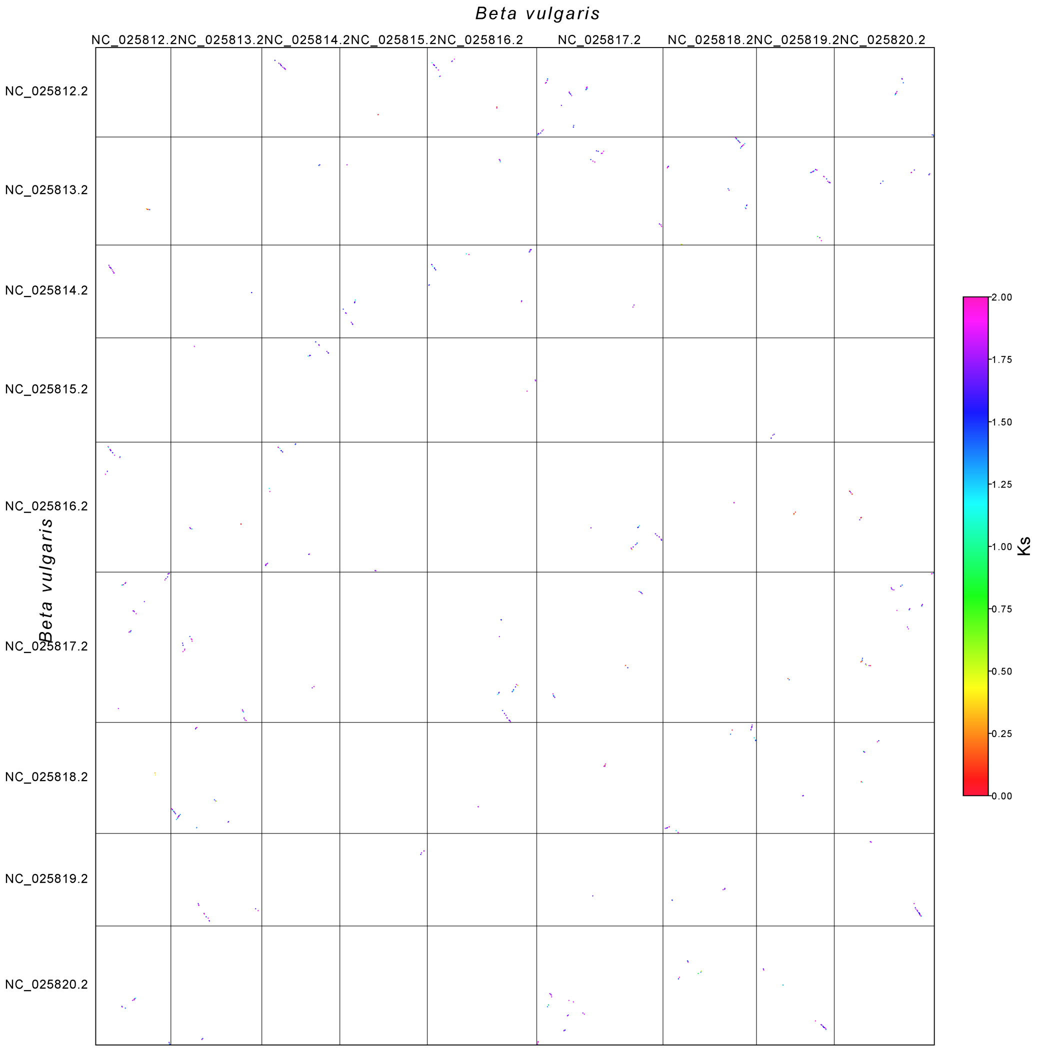

Figure S17. The synteny blocks of the *B. vulgaris* genome.

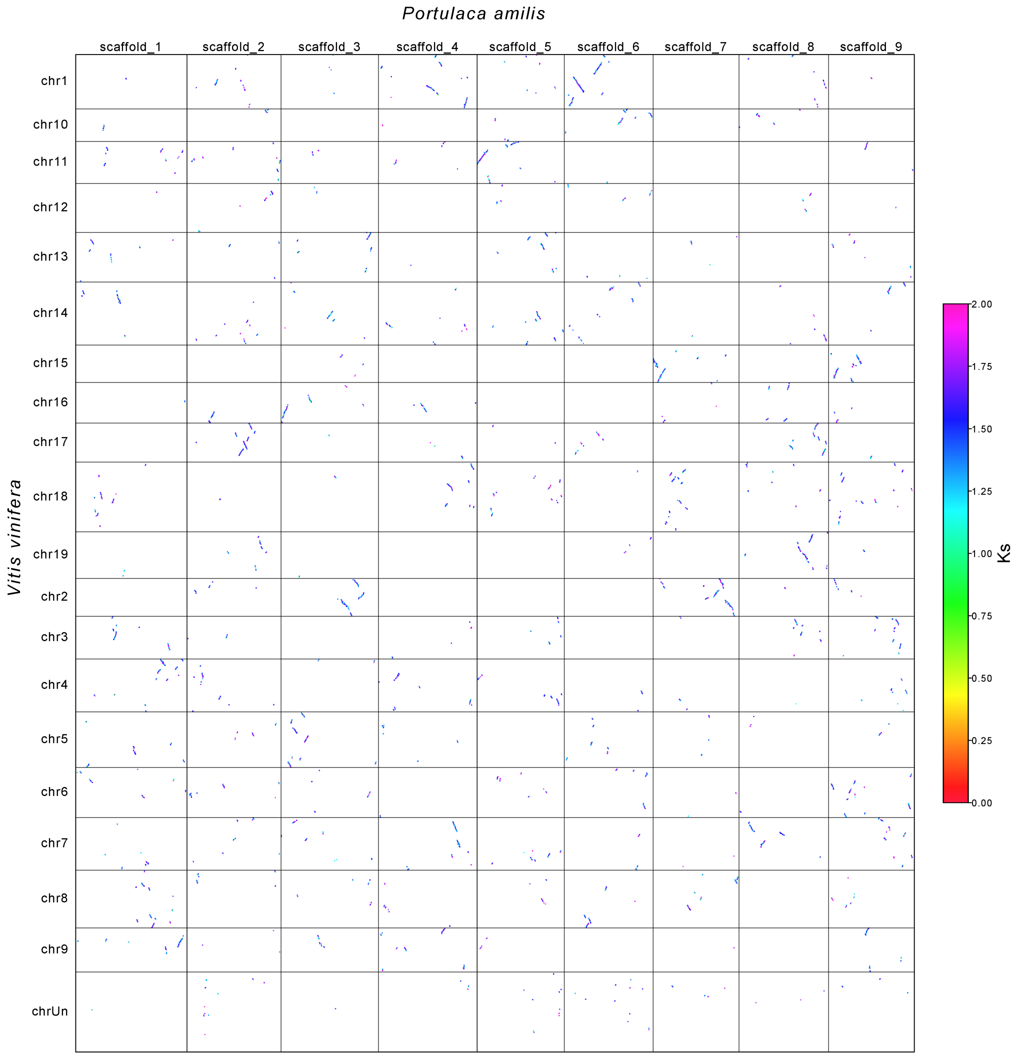

Figure S18. The synteny blocks of the *P. amilis* compared with *V. vinifera* genome.

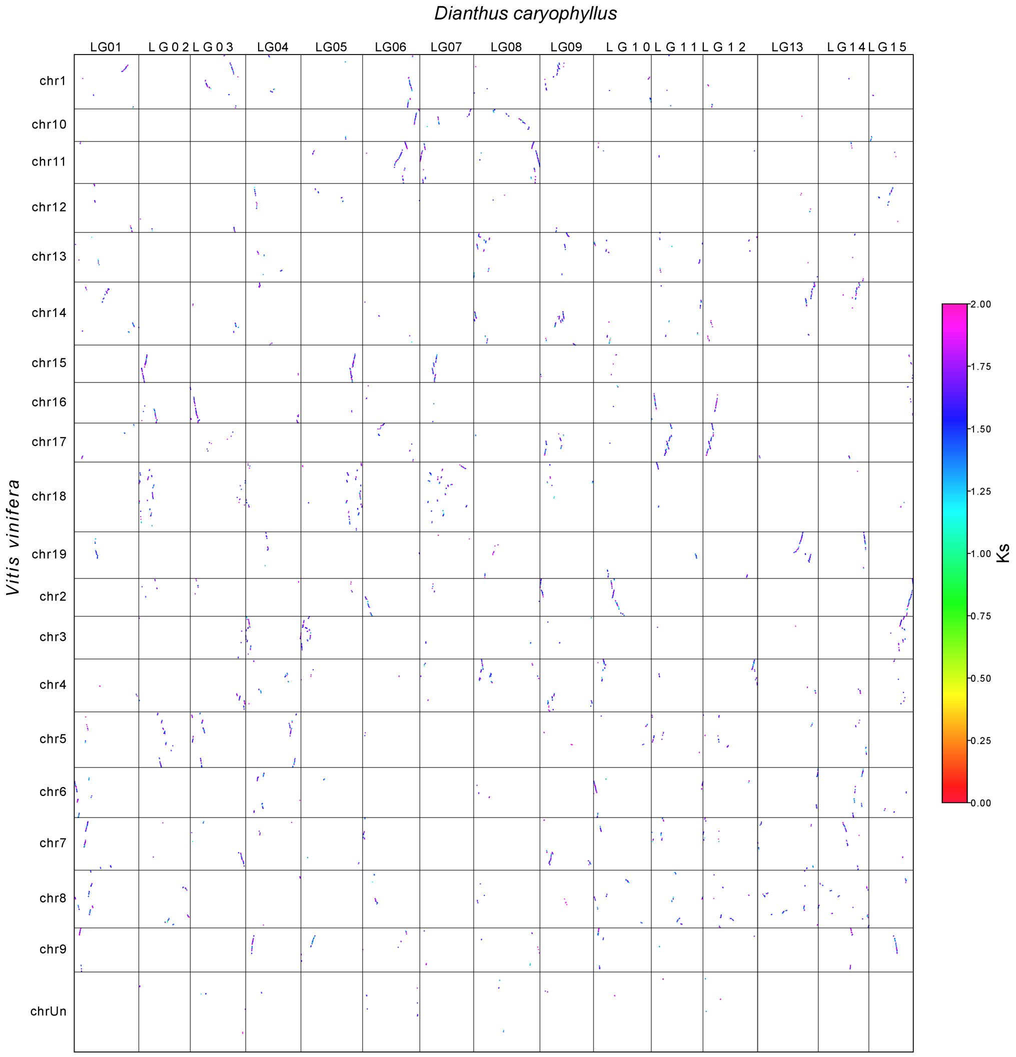

Figure S19. The synteny blocks of the *D. caryophyllus* compared with *V. vinifera* genome.

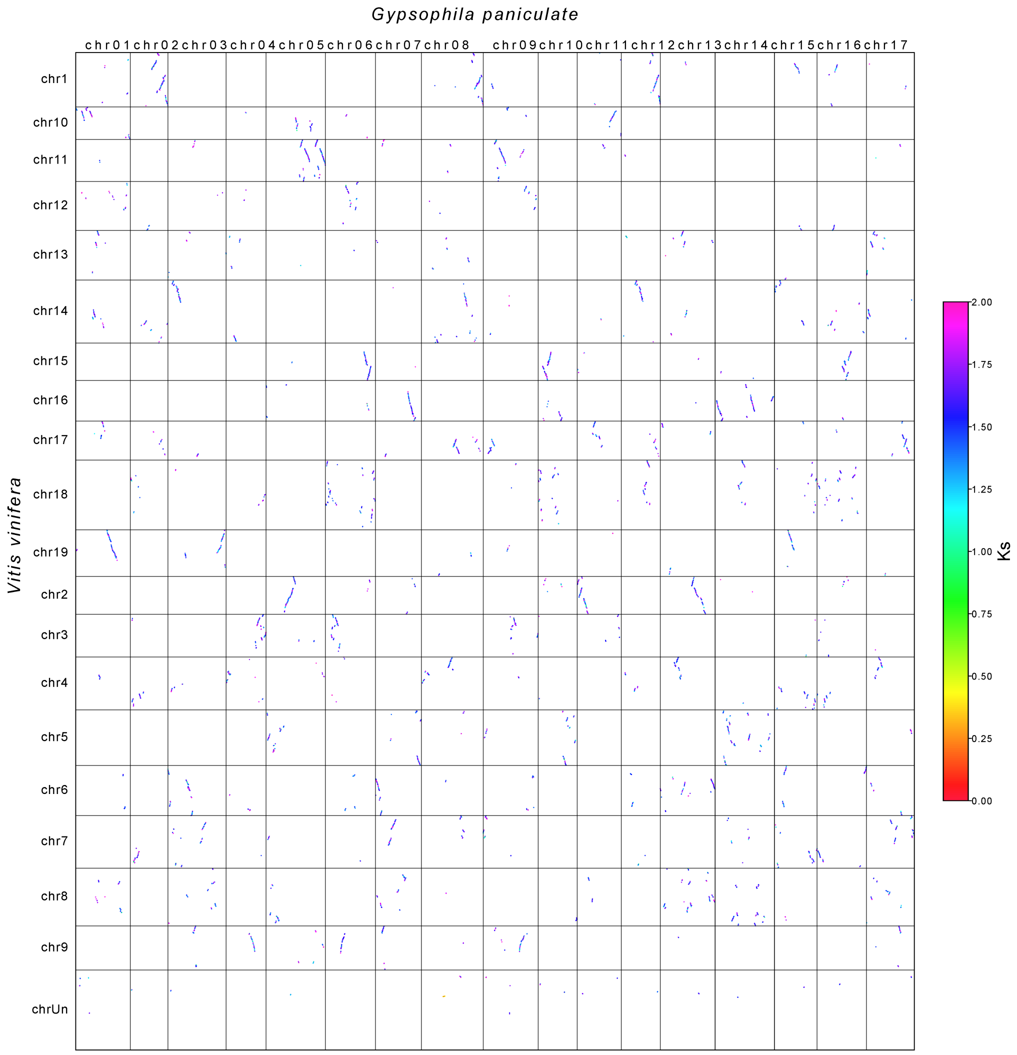

Figure S20. The synteny blocks of the *G. paniculate* compared with *V. vinifera* genome.

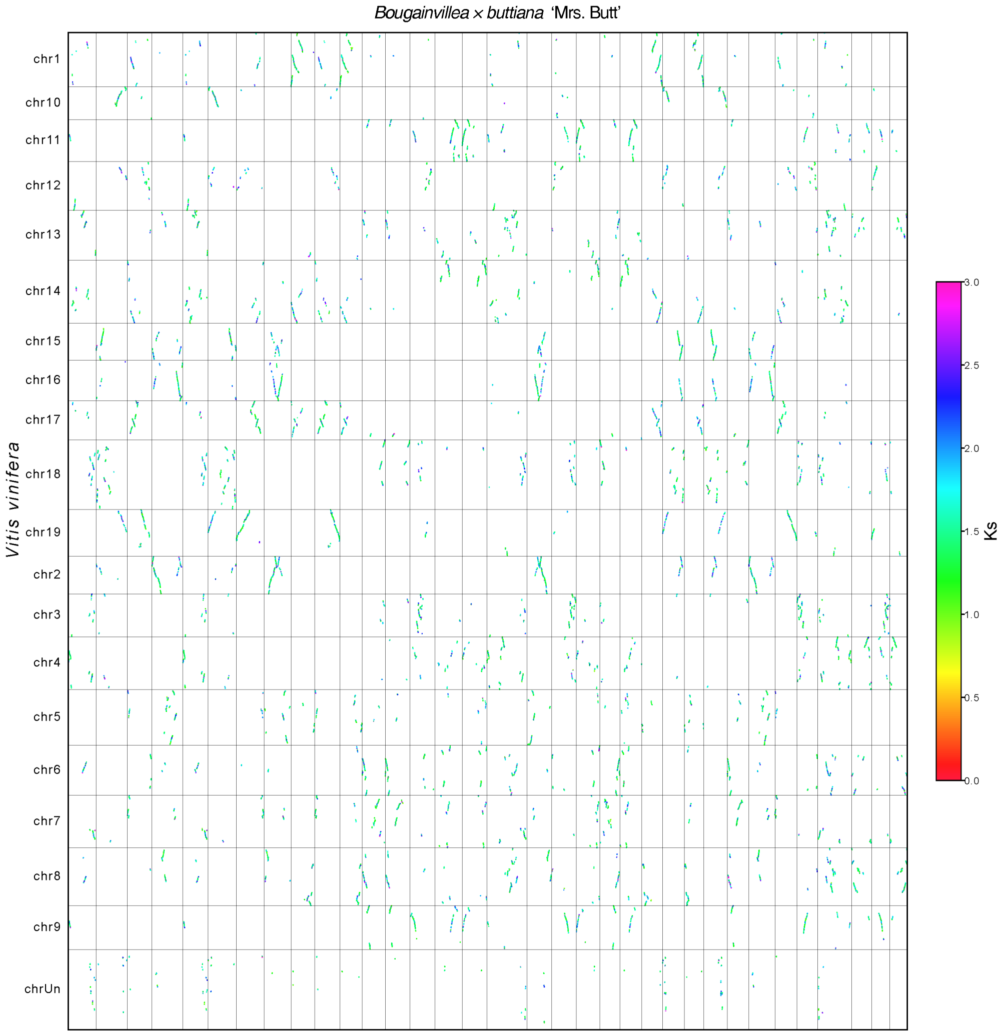

Figure S21. The synteny blocks of the *B.* × *buttiana* 'Mrs. Butt' compared with *V. vinifera* genome.

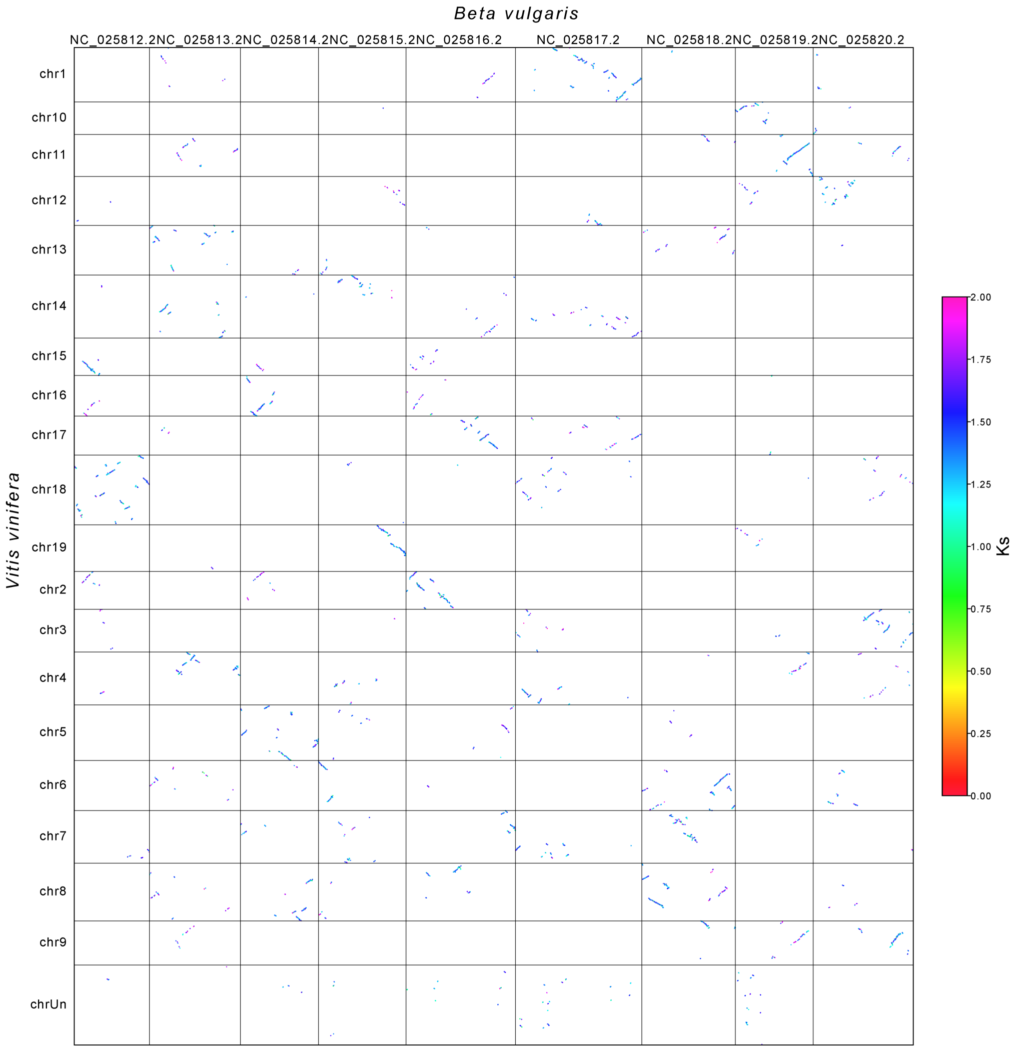

Figure S22. The synteny blocks of the *B. vulgaris* compared with *V. vinifera* genome.

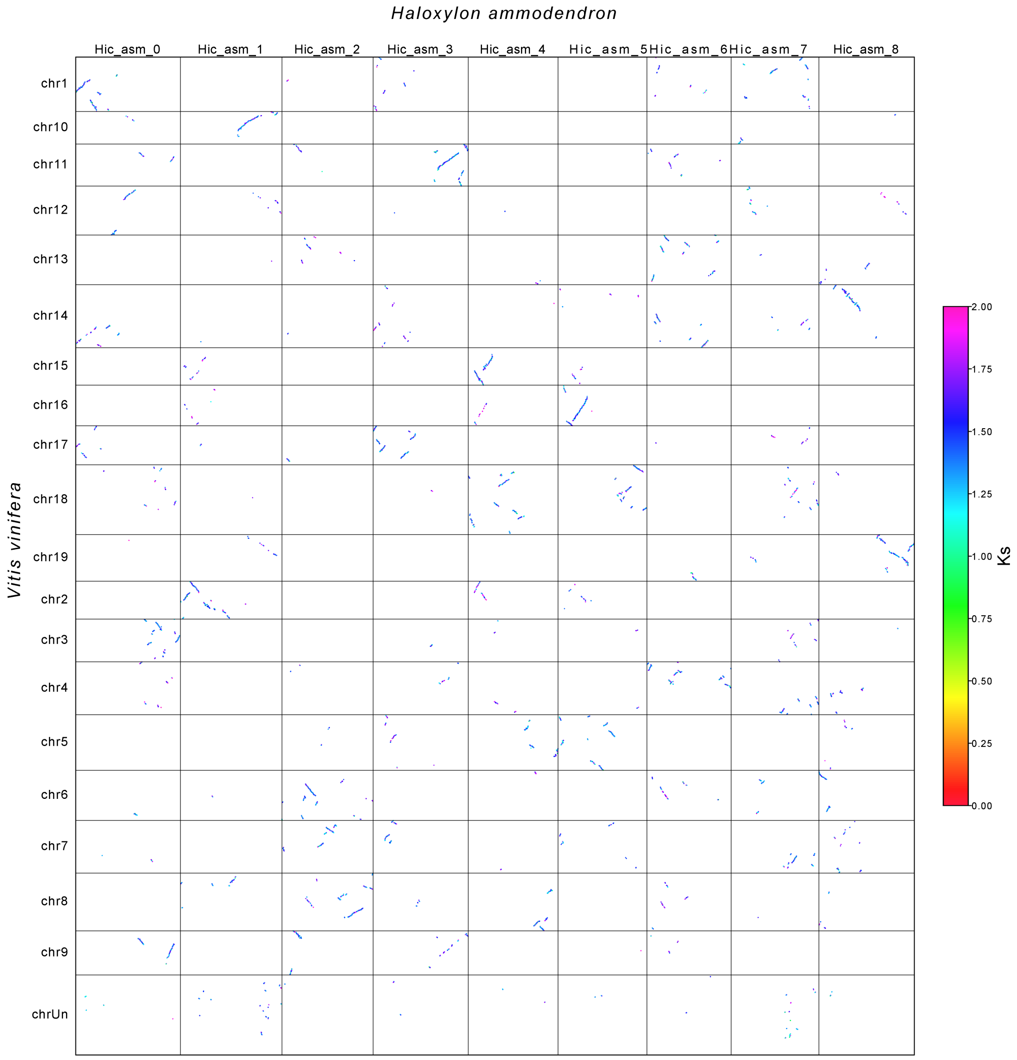

Figure S23. The synteny blocks of the *H. ammodendron* compared with *V. vinifera* genome.

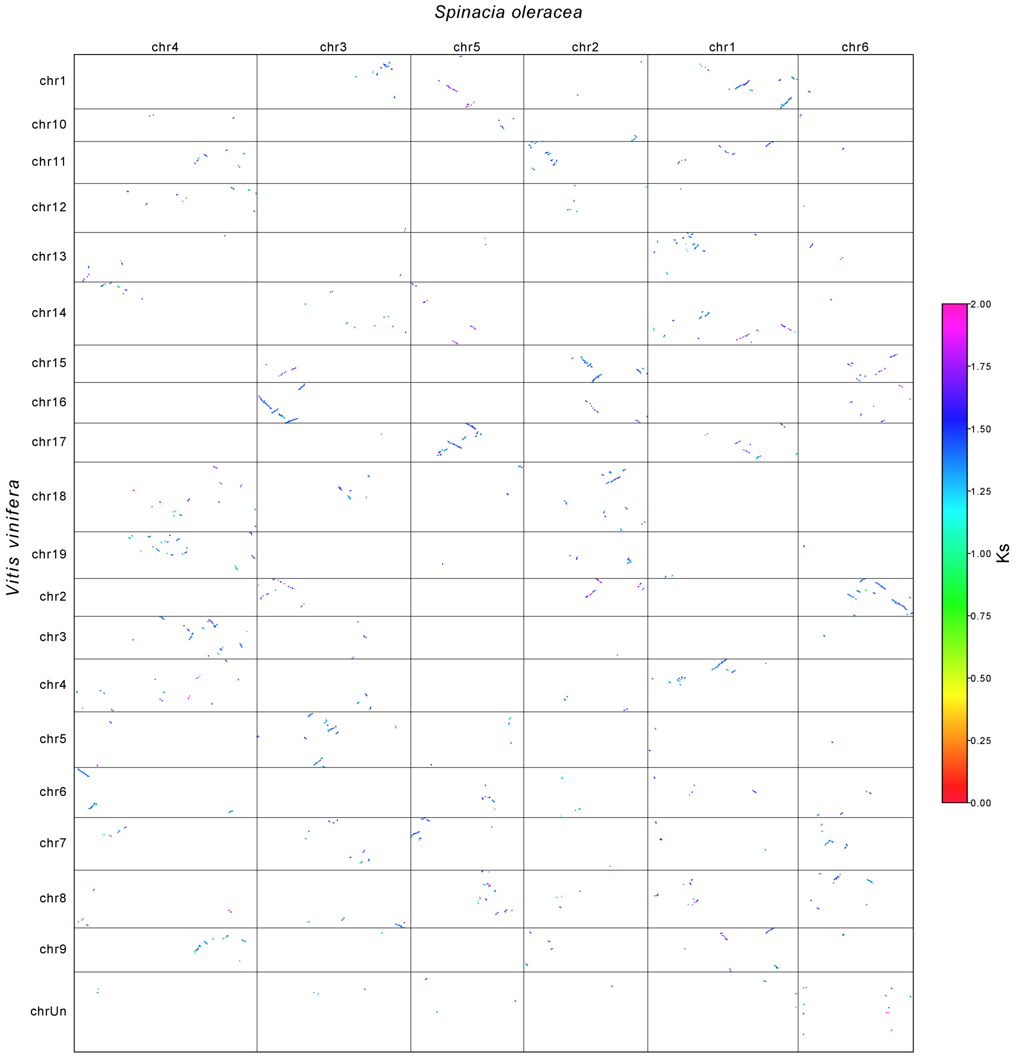

Figure S24. The synteny blocks of the *S. oleracea* compared with *V. vinifera* genome.

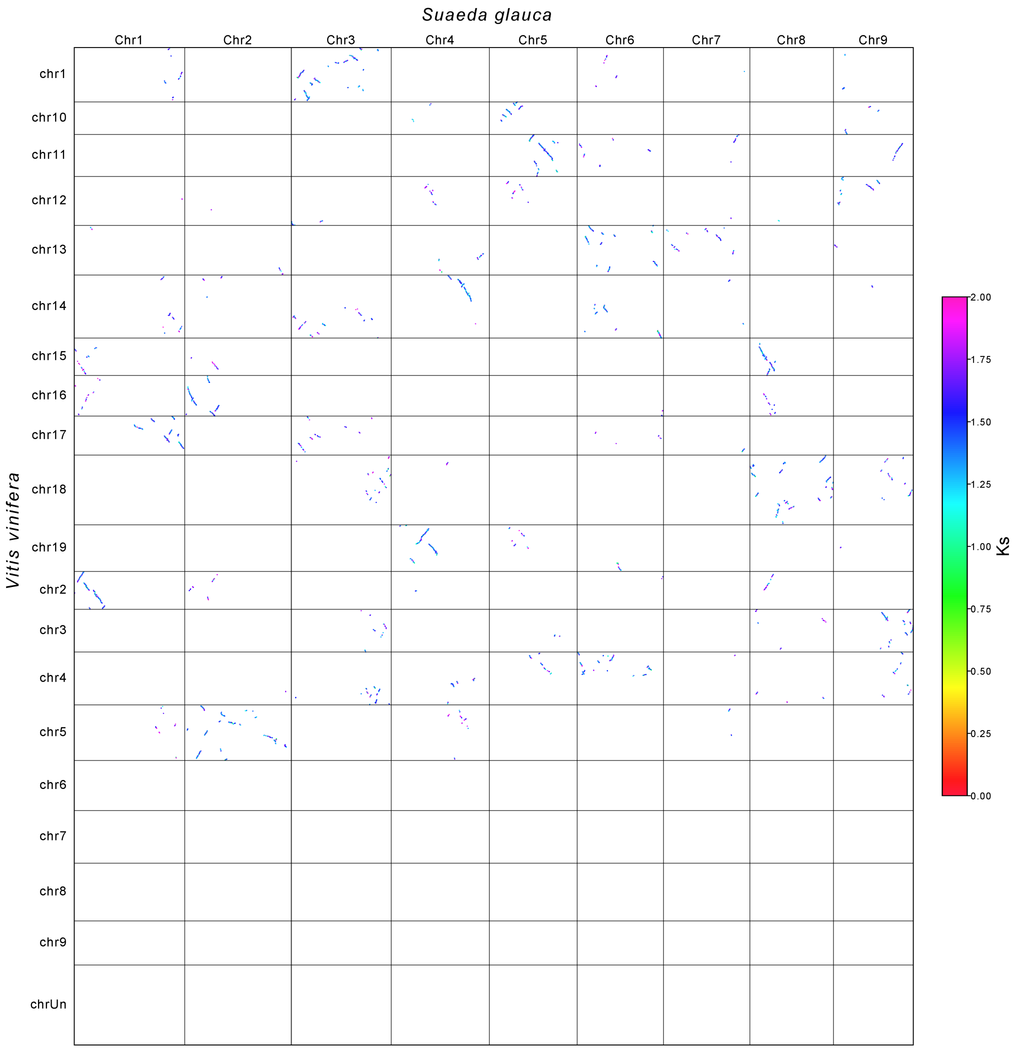

Figure S25. The synteny blocks of the *S. glauca* compared with *V. vinifera* genome.

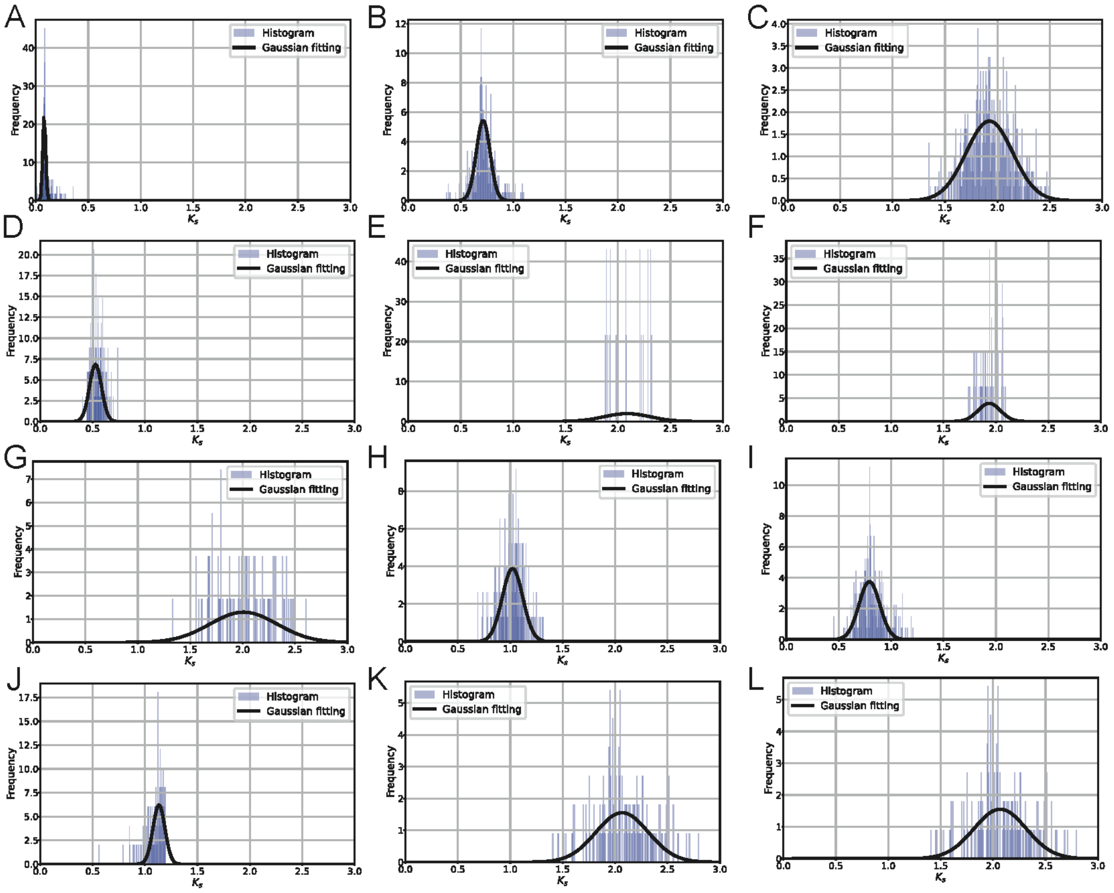

Figure S26. Distribution of Ks of intragenomic syntenic blocks of each species. The histogram showed the raw Ks distribution and the black curve represent the Gaussian fit of the raw Ks. All the peaks correspond to Figure 2A in the main text. A, B and C for *B.* × *buttiana* ‘Mrs. Butt’, D and E for *A. cruentus*, F for *B. vulgaris*, G for *H. ammodendron*, H for *D. caryophyllus*, I for *G. paniculate*, J for *P. amilis*, K for *S. glauca* and L for *S. oleracea*.

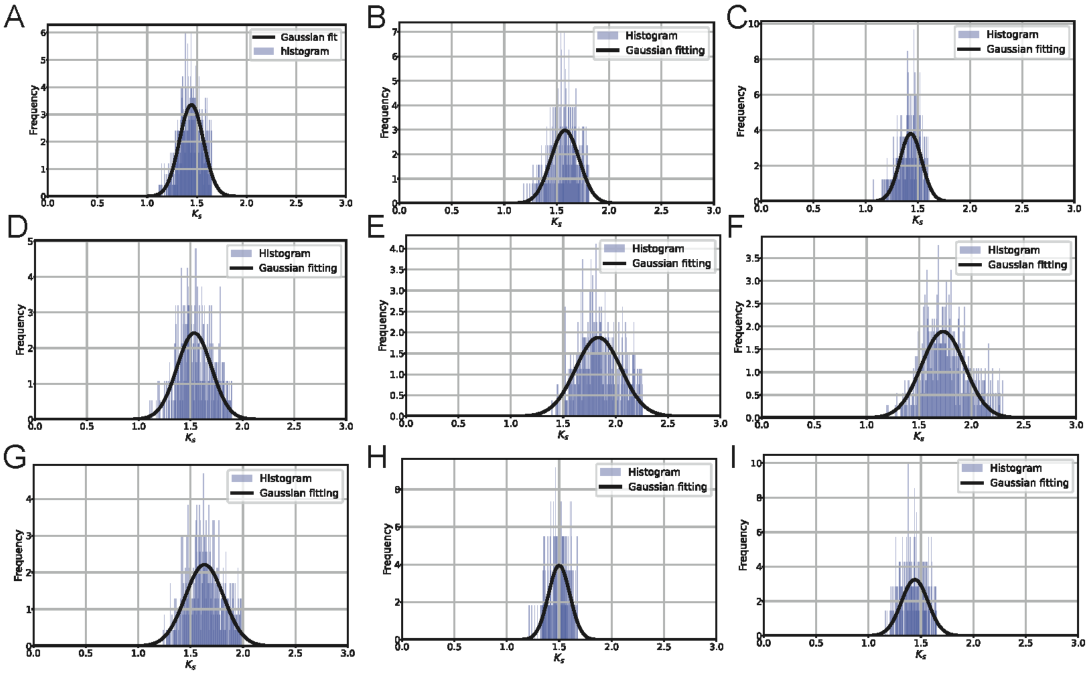

Figure S27. Distribution of Ks of intergenomic syntenic blocks of each species compared with *V. vinifera*. The histogram showed the raw Ks distribution and the black curve represent the Gaussian fit of the raw Ks. All the peaks correspond to Figure 2B in the main text. A for *B.* × *buttiana* 'Mrs. Butt', B for *A. cruentus*, C for *B. vulgaris*, D for *H. ammodendron*, E for *D. caryophyllus*, F for *G. paniculate*, G for *P. amilis*, H for *S. glauca* and I for *S. oleracea*.

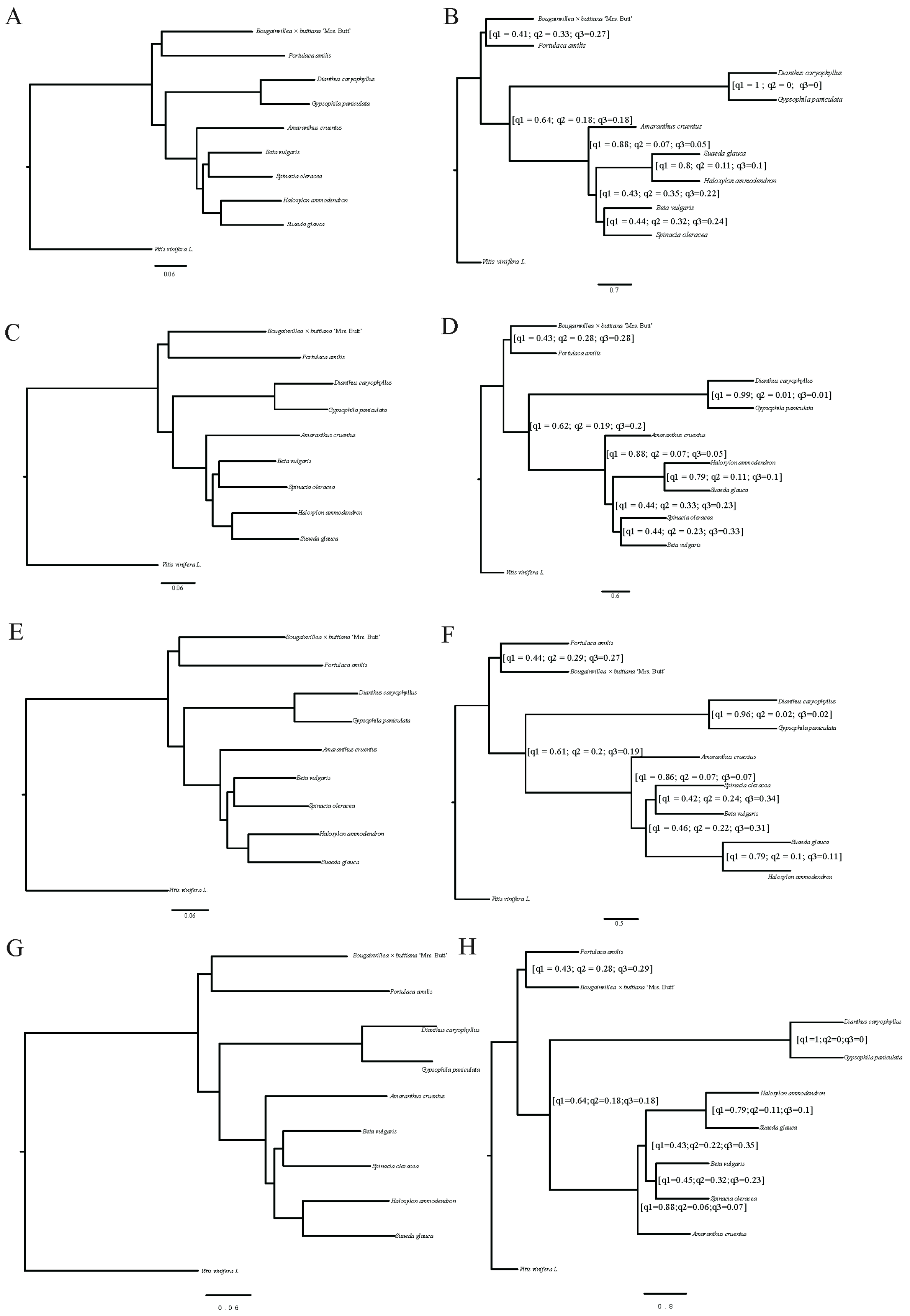
A
Bougainvillea × buttiana 'Mrs. Butt'
Portulaca amilis
Dianthus caryophyllus
Gypsophila paniculata
Amaranthus cruentus
Beta vulgaris
Spinacia oleracea
Haloxylon ammodendron
Suaeda glauca
Vitis vinifera L.
0.06
B
Bougainvillea × buttiana 'Mrs. Butt'
[q1 = 0.41; q2 = 0.33; q3=0.27]
Portulaca amilis
Dianthus caryophyllus
[q1 = 1 ; q2 = 0; q3=0]
Gypsophila paniculata
[q1 = 0.64; q2 = 0.18; q3=0.18]
Amaranthus cruentus
[q1 = 0.88; q2 = 0.07; q3=0.05]
Suaeda glauca
[q1 = 0.8; q2 = 0.11; q3=0.1]
Haloxylon ammodendron
[q1 = 0.43; q2 = 0.35; q3=0.22]
Beta vulgaris
[q1 = 0.44; q2 = 0.32; q3=0.24]
Spinacia oleracea
Vitis vinifera L.
0.7
C
Bougainvillea × buttiana 'Mrs. Butt'
Portulaca amilis
Dianthus caryophyllus
Gypsophila paniculata
Amaranthus cruentus
Beta vulgaris
Spinacia oleracea
Haloxylon ammodendron
Suaeda glauca
Vitis vinifera L.
0.06
D
Bougainvillea × buttiana 'Mrs. Butt'
[q1 = 0.43; q2 = 0.28; q3=0.28]
Portulaca amilis
Dianthus caryophyllus
[q1 = 0.99; q2 = 0.01; q3=0.01]
Gypsophila paniculata
[q1 = 0.62; q2 = 0.19; q3=0.2]
Amaranthus cruentus
[q1 = 0.88; q2 = 0.07; q3=0.05]
Haloxylon ammodendron
[q1 = 0.79; q2 = 0.11; q3=0.1]
Suaeda glauca
[q1 = 0.44; q2 = 0.33; q3=0.23]
Spinacia oleracea
[q1 = 0.44; q2 = 0.23; q3=0.33]
Beta vulgaris
Vitis vinifera L.
0.6
E
Bougainvillea × buttiana 'Mrs. Butt'
Portulaca amilis
Dianthus caryophyllus
Gypsophila paniculata
Amaranthus cruentus
Beta vulgaris
Spinacia oleracea
Haloxylon ammodendron
Suaeda glauca
Vitis vinifera L.
0.06
F
Portulaca amilis
[q1 = 0.44; q2 = 0.29; q3=0.27]
Bougainvillea × buttiana 'Mrs. Butt'
Dianthus caryophyllus
[q1 = 0.96; q2 = 0.02; q3=0.02]
Gypsophila paniculata
[q1 = 0.61; q2 = 0.2; q3=0.19]
Amaranthus cruentus
[q1 = 0.86; q2 = 0.07; q3=0.07]
Spinacia oleracea
[q1 = 0.42; q2 = 0.24; q3=0.34]
Beta vulgaris
[q1 = 0.46; q2 = 0.22; q3=0.31]
Suaeda glauca
[q1 = 0.79; q2 = 0.1; q3=0.11]
Haloxylon ammodendron
Vitis vinifera L.
0.5
G
Bougainvillea × buttiana 'Mrs. Butt'
Portulaca amilis
Dianthus caryophyllus
Gypsophila paniculata
Amaranthus cruentus
Beta vulgaris
Spinacia oleracea
Haloxylon ammodendron
Suaeda glauca
Vitis vinifera L.
0.06
H
Portulaca amilis
[q1 = 0.43; q2 = 0.28; q3=0.29]
Bougainvillea × buttiana 'Mrs. Butt'
Dianthus caryophyllus
[q1=1;q2=0;q3=0]
Gypsophila paniculata
[q1=0.64;q2=0.18;q3=0.18]
Haloxylon ammodendron
[q1=0.79;q2=0.11;q3=0.1]
Suaeda glauca
[q1=0.43;q2=0.22;q3=0.35]
Beta vulgaris
[q1=0.45;q2=0.32;q3=0.23]
Spinacia oleracea
[q1=0.88;q2=0.06;q3=0.07]
Amaranthus cruentus
Vitis vinifera L.
0.8

Figure S28. The different species tree generated by different data sets and different methods. For A and B used the dataset of SSC, A was generated by the supermatrix from the IQTREE, B was generated by the single gene tree from the ASTRAL; for C and D used the dataset of LC2, C was generated by the supermatrix form the IQTREE, D was generated by the single tree from the ASTRAL; for E and F used the dataset of LC5, E was generated by the supermatrix from the IQTREE, F was generated by the single tree from the ASTRAL; for G and H used the dataset of SCG, G was generated by the supermatrix from the IQTREE, H was generated from the ASTRAL. All bootstrap values were 100.

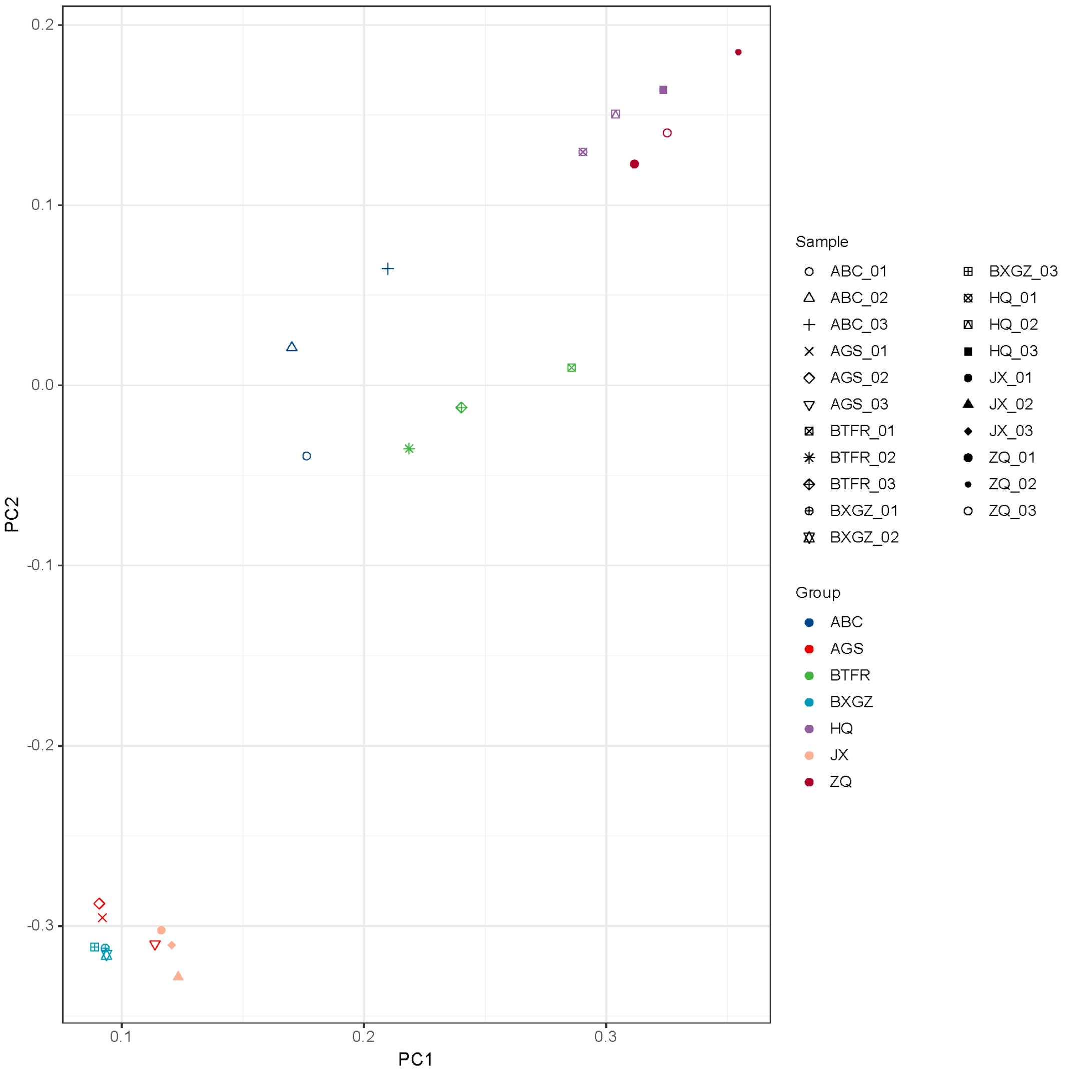

Figure S29. The PCA analysis of the transcriptome data of the total genes' FPKM used in this study. The samples of ABC, AGS, BTFR, BXGZ, HQ, JX, ZQ referred to *B. hybrid* 'Sundance' (ABC), *B. hybrid* 'Elizabeth Angus' (AGC), *B.* × *buttiana* 'Mrs. Butt' (BTFR), *B. hybrid* 'Mrs. Eva White' (BXGZ), *B. hybrid* 'Firecracker Yellow' (HQ), *B. hybrid* 'Rijnstar Pink' (JX) and *B. hybrid* 'Firecracker Purple' (ZQ). The number of 01, 02 and 03 represent the biological repetition number.

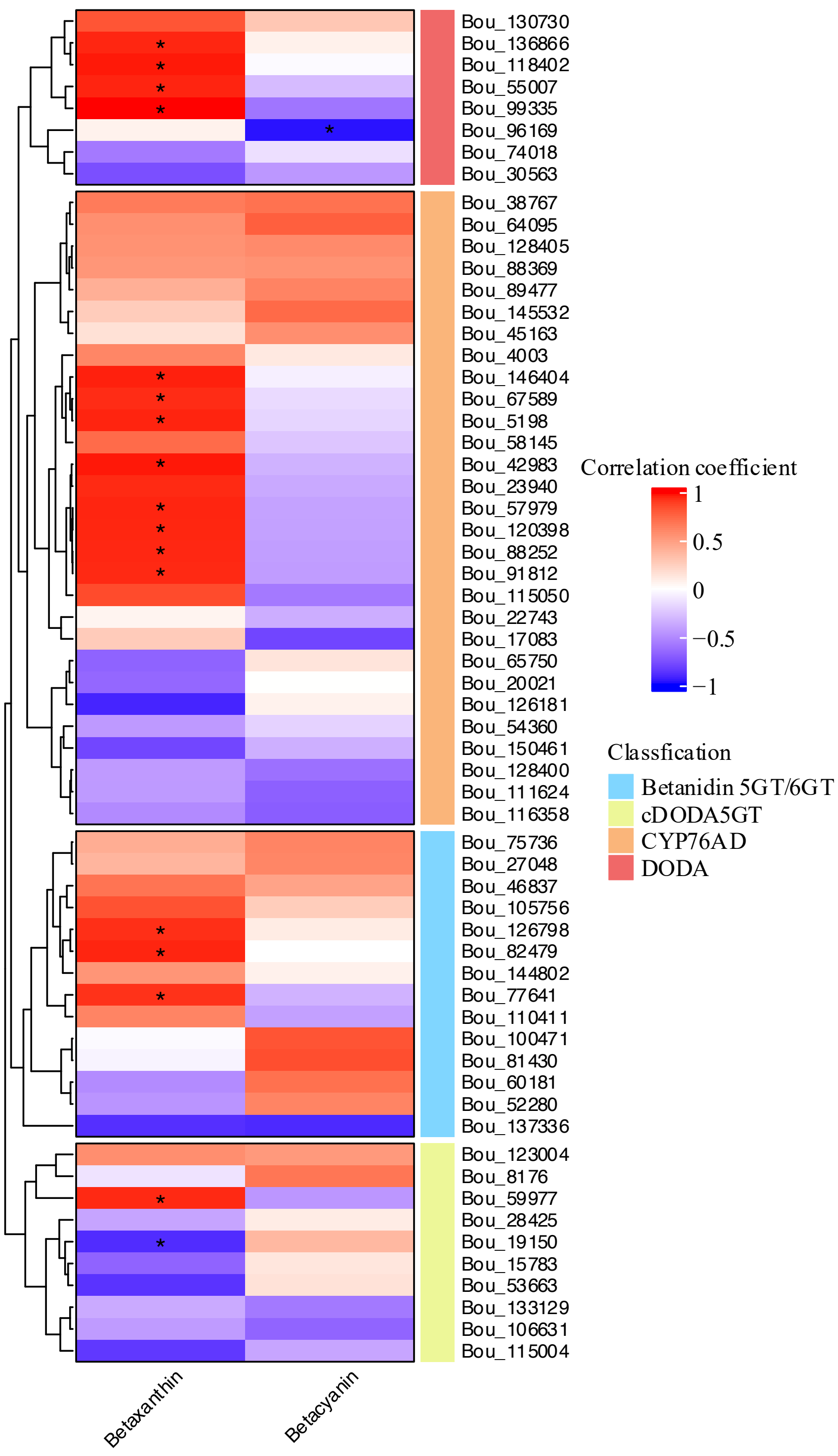

Figure 30. The correlation heatmap between the betalains biosynthetic genes' FPKM and betalains content through ZQ, HQ, AGS, JX and BXGZ. The '*' marked means the gene exhibited a significant correlation with the certain component.
